# Unraveling *Enterococcus* susceptibility to quaternary ammonium compounds: genes, phenotypes, and the impact of environmental conditions

**DOI:** 10.1128/spectrum.02324-23

**Published:** 2023-09-22

**Authors:** Ana P. Pereira, Patrícia Antunes, Paula Bierge, Rob J. L. Willems, Jukka Corander, Teresa M. Coque, Oscar Q. Pich, Luisa Peixe, Ana R. Freitas, Carla Novais, from the ESCMID Study Group on Food- and Water-borne Infections (EFWISG)

**Affiliations:** 1 UCIBIO-Applied Molecular Biosciences Unit, Laboratory of Microbiology, Faculty of Pharmacy, University of Porto, Porto, Portugal; 2 Associate Laboratory i4HB - Institute for Health and Bioeconomy, Laboratory of Microbiology, Faculty of Pharmacy, University of Porto, Porto, Portugal; 3 Faculty of Nutrition and Food Sciences, University of Porto, Porto, Portugal; 4 Laboratori de Recerca en Microbiologia i Malalties Infeccioses, Parc Taulí Hospital Universitari, Institut d’Investigació i Innovació Parc Taulí (I3PT-CERCA), Universitat Autònoma de Barcelona, Sabadell, Spain; 5 Institut de Biotecnologia i Biomedicina, Universitat Autònoma de Barcelona, Bellaterra, Spain; 6 Department of Medical Microbiology, University Medical Center Utrecht, Utrecht, the Netherlands; 7 Department of Biostatistics, Faculty of Medicine, University of Oslo, Oslo, Norway; 8 Parasites and Microbes, Wellcome Sanger Institute, Cambridge, UK; 9 Department of Mathematics and Statistics, Helsinki Institute of Information Technology, University of Helsinki, Helsinki, Finland; 10 Servicio de Microbiologia, Hospital Universitario Ramón y Cajal, Madrid, Spain; 11 Centro de Investigación Biomédica en Enfermedades Infecciosas (CIBERINFEC), Madrid, Spain; 12 1H-TOXRUN, One Health Toxicology Research Unit, University Institute of Health Sciences, CESPU, CRL., Gandra, Portugal; University at Albany, Albany, New York, USA

**Keywords:** Bacillota, biocide, disinfection, One Health

## Abstract

Quaternary ammonium compounds (QACs) have been extensively used in the community, healthcare facilities, and food chain, in concentrations between 20 and 30,000 mg/L. *Enterococcus faecalis* and *Enterococcus faecium* are ubiquitous in these settings and are recognized as nosocomial pathogens worldwide, but QACs’ activity against strains from diverse epidemiological and genomic backgrounds remained largely unexplored. We evaluated the role of *Enterococcus* isolates from different sources, years, and clonal lineages as hosts of QACs tolerance genes and their susceptibility to QACs in optimal, single-stress and cross-stress growth conditions. Only 1% of the *Enterococcus* isolates included in this study and 0.5% of publicly available *Enterococcus* genomes carried *qacA/B, qacC, qacG, qacJ, qacZ, qrg, bcrABC* or *oqxAB* genes, shared with >60 species of Bacillota, Pseudomonadota, Actinomycetota, or Spirochaetota. These genes were generally found within close proximity of antibiotics and/or metals resistance genes. The minimum inhibitory concentrations (MIC) and minimum bactericidal concentrations (MBC) of benzalkonium chloride (BC) and didecyldimethylammonium chloride ranged between 0.5 and 4 mg/L (microdilution: 37°C/20 h/pH = 7/aerobiosis) for 210 *E. faecalis* and *E. faecium* isolates (two isolates carrying *qacZ*). Modified growth conditions (e.g., 22°C/pH = 5) increased MIC_BC_/MBC_BC_ (maximum of eightfold and MBC_BC_ = 16 mg/L) and changed bacterial growth kinetics under BC toward later stationary phases in both species, including in isolates without QACs tolerance genes. In conclusion, *Enterococcus* are susceptible to in-use QACs concentrations and rarely carry QACs tolerance genes. However, their potential gene exchange with different microbiota, the decreased susceptibility to QACs under specific environmental conditions, and the presence of subinhibitory QACs concentrations in various settings may contribute to the selection of particular strains and, thus, require a One Health strategy to maintain QACs effectiveness.

**IMPORTANCE:**

Despite the increasing use of quaternary ammonium compounds (QACs), the susceptibility of pathogens to these antimicrobials remains largely unknown. *Enterococcus faecium* and *Enterococcus faecalis* are susceptible to in-use QACs concentrations and are not main hosts of QACs tolerance genes but participate in gene transfer pathways with diverse bacterial taxa exposed to these biocides. Moreover, QACs tolerance genes often share the same genetic contexts with antibiotics and/or metals resistance genes, raising concerns about potential co-selection events. *E. faecium* and *E. faecalis* showed increased tolerance to benzalkonium chloride under specific environmental conditions (22°C, pH = 5), suggesting that strains might be selected in settings where they occur along with subinhibitory QACs concentrations. Transcriptomic studies investigating the cellular mechanisms of *Enterococcus* adaptation to QACs tolerance, along with longitudinal metadata analysis of tolerant populations dynamics under the influence of diverse environmental factors, are essential and should be prioritized within a One Health strategy.

## INTRODUCTION

*Enterococcus* spp. are major opportunistic pathogens of humans and animals that easily spread through contaminated abiotic surfaces, enhancing the risk of transmission between different hosts (1, 2). They are widely distributed in various environments, including foods, water systems, and soil and serve as indicators of fecal contamination due to their ability to withstand harsh conditions and different stresses. Additionally, they are known to engage in horizontal genetic transfer with local microbiota (1 – 3). Despite the significant increase in the usage of biocides, such as quaternary ammonium compounds (QACs), in recent years, and their key role in controlling the spread of pathogens, the susceptibility of major opportunistic pathogens, including *Enterococcus* spp., to these antimicrobials remains largely unknown (4, 5).

QACs are cationic biocides used extensively as disinfectants, antiseptics, and preservatives in human and veterinary healthcare facilities, food production settings, and consumer products (e.g., hygiene products, eye drops, and mouthwashes) (5 – 7). Among the most used are benzalkonium chloride (BC) and didecyldimethylammonium chloride (DDAC) (5, 8). Their action may be bacteriostatic or bactericidal depending on the concentration used, and it involves the loss of the membrane’s physical and ionic integrity, with leakage of cytoplasmic components, osmotic dysregulation, inhibition of respiratory enzymes and transport, and oxidative stress (6, 8, 9). In-use concentrations of QACs range from 20 to 30,000 mg/L (7, 10). Diverse concentration gradients (less than 0.001 to 6 mg/L) have also been found in sewage and surface water and soil/sediments, after domestic, hospital, and industrial (including food processing) discharges, either directly or via wastewater treatment systems, followed by dilution in the environment and biodegradation (6, 7, 11). Insufficient cleaning prior to disinfection or inadequate application of the biocide (e.g., dosage error, excess of organic matter, or the use of cotton and microfiber cloths that greatly reduce QACs concentration) in the clinical or food processing settings may also contribute to QACs use in subinhibitory concentrations in these particular contexts (8, 12). Thus, diverse microbiomes are often exposed to a wide range of QACs concentrations that may result in changes in their taxonomic composition and promote the selection of populations with decreased susceptibility to QACs and other antimicrobial compounds (biocides or antibiotics) by a co- or cross-selection process (7, 13 – 16). When the in-use concentrations of a biocide remain higher than the minimum inhibitory concentrations (MIC) or minimum bactericidal concentrations (MBC) observed *in vitro*, a decreased susceptibility to biocides is often described as “tolerance” (the term used in this manuscript) (17 – 19). On the contrary, the term “resistance” implies that the microorganisms are not inactivated by the in-use concentrations of a biocide (17 – 19). Tolerance to QACs is mainly associated with the acquisition or overexpression of genes encoding efflux pumps, although adaptive tolerance mechanisms such as changes in the cell membrane composition have also been reported (6, 19).

QACs efflux determinants belonging to the MFS (major facilitator superfamily; e.g., *qacA/B*), SMR (small multidrug resistance; e.g., *qacC, qacZ, qrg, bcrABC*), and RND (resistance nodulation division; e.g., *oqxAB*) families have been detected in *Enterococcus* spp. (20 – 26). However, few studies have addressed their susceptibility and genomic adaptation to QACs (10, 20, 21, 26, 27), especially within a comprehensive context of *Enterococcus* population structure, sources, and time spans. In fact, the role of QACs tolerance genes on *Enterococcus* adaptation to these compounds remains elusive due to contradictory data regarding the increase of QACs tolerance in strains carrying such genes (20 – 22, 24). This discrepancy can be attributed, in part, to the absence of standardized methodologies and universally accepted epidemiological cutoffs (28). Of note, most studies assessing the susceptibility of *Enterococcus* and other bacteria to QACs typically conduct tests under ideal standardized growth conditions. Thus, the impact of various physicochemical challenges present in diverse natural ecosystems, such as pH, temperature, and other pollutants, remains largely unknown (29).

In this study, we aimed to assess the occurrence of known transferable QACs tolerance genes in *Enterococcus* spp. isolates and public genomes and their flow between *Enterococcus* and other bacterial taxa. Also, we evaluated the activity of BC and DDAC in different growth conditions, among a collection of phylogenetically diverse *E. faecalis* and *E. faecium* from different sources, time spans, and antibiotic resistance profiles.

## RESULTS

### *Enterococcus* carry broad-host QACs tolerance genes widely spread across different bacterial taxa

The presence of QACs tolerance genes was investigated in *Enterococcus* from diverse epidemiological backgrounds, including 210 *Enterococcus* isolates from human and non-human sources (105 *E. faecium* and 105 *E. faecalis*) and 22,428 genomes publicly available at the NCBI database.

**Fig 1:**
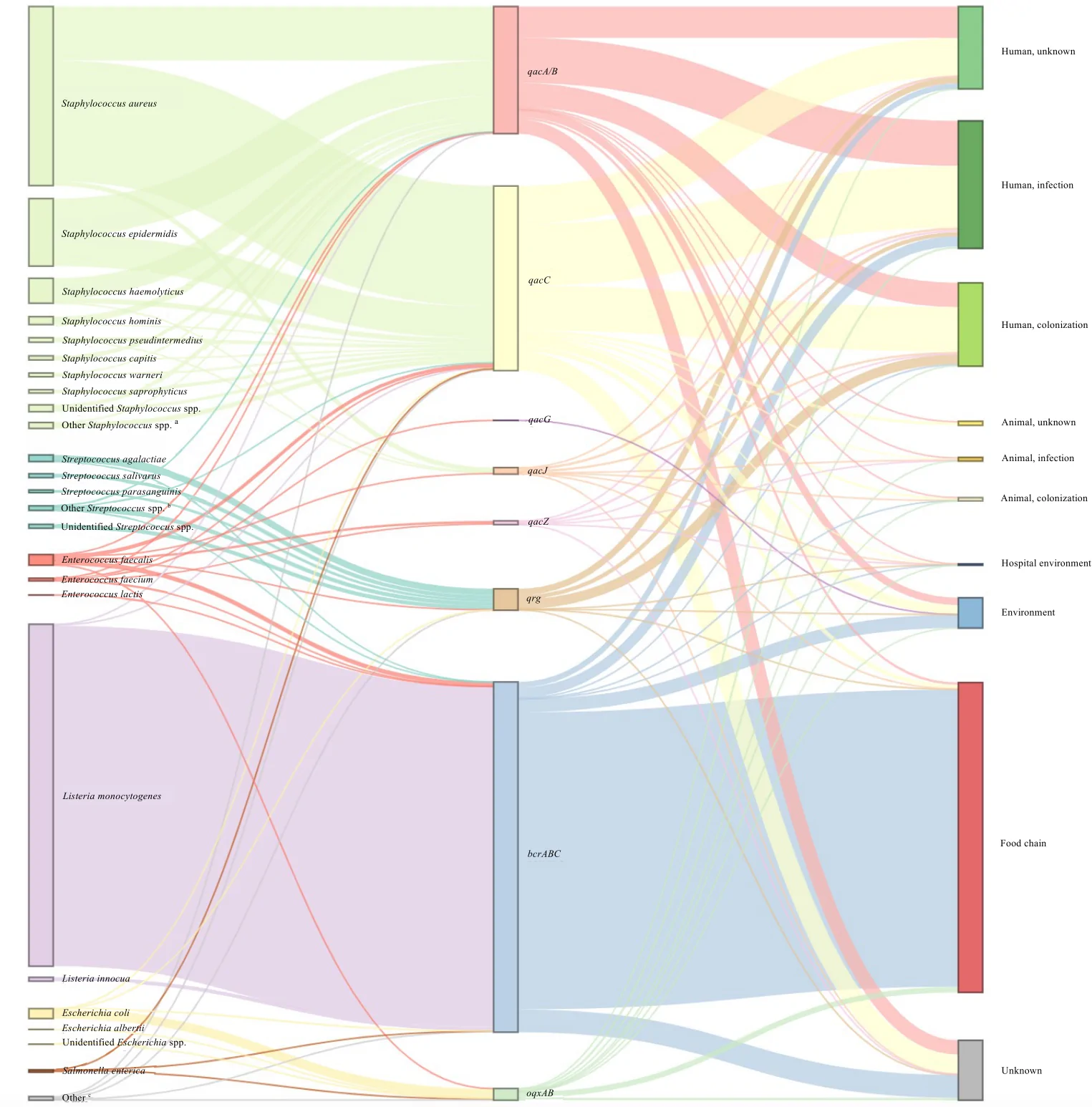
Sankey diagram showing the distribution of QACs tolerance genes found among *Enterococcus* spp. and shared with other bacterial taxa. Using the blastp tool, the following genes were searched in *Enterococcus* spp. genomes and proteins available at the NCBI database: *qacA/B, qacC, qacG, qacJ, qacZ, qrg, bcrABC, oqxAB* (GenBank accession no. HE579074.1, GQ900434.1, Y16944.1, NG_048046.1, KM083808.1, HQ663849.2, NZ_CP011399.1, KT716391.1, respectively). Sequences with ≥90% of query-cover and ≥97% identity were considered. Identical proteins (100% coverage and identity) to the ones identified were then searched in other NCBI published bacterial taxa genomes and proteins in order to infer about the genetic flow of these genes. The diagram was constructed in R using the package networkD3 v0.4 (31). ^a^Other *Staphylococcus* spp.*: Staphylococcus argenteus, Staphylococcus arlettae, Staphylococcus borealis, Staphylococcus caprae, Staphylococcus cohnii, Staphylococcus croceilyticus, Staphylococcus equorum, Staphylococcus lugdunensis, Staphylococcus massiliensis, Staphylococcus pasteuri, Staphylococcus petrasii, Staphylococcus pettenkoferi, Staphylococcus schleiferi, Staphylococcus simulans, Staphylococcus singaporensis.* ^b^Other *Streptococcus* spp.*: Streptococcus anginosus, Streptococcus australis, Streptococcus gordonii, Streptococcus mitis, Streptococcus mutans, Streptococcus oralis, Streptococcus pneumoniae, Streptococcus pseudopneumoniae, Streptococcus pyogenes, Streptococcus vestibularis.* ^c^Other: *Acinetobacter baumannii, Burkholderia cepacian, Campylobacter jejuni, Carnobacterium divergens, Clostridioides difficile, Cutibacterium acnes, Haemophilus parainfluenzae, Klebsiella pneumoniae, Leptospira noguchii, Mammaliicoccus sciuri, Mycobacteroides abscessus, Oenococcus oeni, Providencia alcalifaciens, Pseudomonas aeruginosa, Shigella flexneri, Shinella curvata, Streptomyces* sp.*, Vibrio cholerae.*

Only 1% of the isolates, which included one ST17 *E. faecium* and one ST6 *E. faecalis* carrying the *qacZ* gene, were found to carry QACs tolerance genes. Additionally, among the public genomes analyzed, the presence of QACs tolerance genes (*qacA/B*, *qacC*, *qacG*, *qacJ*, *qacZ*, *qrg*, *bcrABC*, and *oqxAB*) was observed in only 0.5% of the genomes (*n* = 117), which encompassed 93 *E. faecalis*, 23 *E. faecium*, and 1 *Enterococcus lactis* genomes (Fig. 1; Table S1). The isolates ST17 *E. faecium*-E241 and ST6 *E. faecalis*-V583 were collected from a hospital sewage (2002) and a hospitalized patient (1987), respectively (21, 30). The 117 *Enterococcus* isolates from public genomes were collected between 1987 and 2020 and belonged to diverse clonal lineages (30 STs among 93 *E. faecalis*; 13 STs among 23 *E. faecium*) and sources (Table S1). Among these, the *qac* and *qrg* genes were mostly detected in humans, including clinical and surveillance isolates (97%, *n* = 69/71, among isolates with identifiable sources) (*P* ≤ 0.01), whereas *bcrABC* and *oqxAB* were more prevalent in *Enterococcus* strains from the food chain environment (68%, *n* = 28/41) (*P* ≤ 0.01) (Fig. 1; Table S1).

QACs tolerance genes identical to the ones found in *Enterococcus* strains were shared with bacterial species from Bacillota, Pseudomonadota, Actinomycetota, and Spirochaetota (former Firmicutes, Proteobacteria, Actinobacteria, and Spirochaetes, respectively) phyla (Fig. 1) (32). The *qacA/B*, *qacC,* and *qacJ* were detected in 20 genera (20, 38, and 4 species, respectively), mainly in *Staphylococcus* spp. (97%, *n* = 2,691/2,777, among bacteria carrying *qac* genes) (*P* ≤ 0.01) and particularly in *S. aureus* (58%, *n* = 1,565/2,691 of the *Staphylococcus* genomes carrying *qac* genes) (Fig. 1). The predominant genera sharing an identical *qrg* with *Enterococcus* strains was *Streptococcus* sp. (98%, *n* = 183/187) (*P* ≤ 0.01), among the five genera (14 species) in which the gene was found. The *qacG* and *qacZ* were only found in *Enterococcus*, according to our sequence selection criteria. As observed for *Enterococcus* isolates, most strains carrying *qac* and *qrg* genes had a human origin (88%, *n* = 2,387/2,699, among isolates with identifiable sources) (*P* ≤ 0.01) (Fig. 1). The *bcrABC* gene cluster was identified in six genera (10 species), and *oqxAB* gene cluster was identified in four genera (five species), mainly in *Listeria monocytogenes* (97%, *n* = 2,975/3,060, among those carrying *bcrABC*) (*P* ≤ 0.01) and *Escherichia coli* (78%, *n* = 80/102, among those carrying *oqxAB*) (*P* ≤ 0.01), respectively (Fig. 1). Of note, *bcrABC* and *oqxAB* were, as mentioned above for enterococci, more prevalent in food chain isolates (84%, *n* = 2,645/2,942) (*P* ≤ 0.01) (Fig. 1).

### Genetic contexts of QACs tolerance genes are diverse and enriched in antimicrobial resistance genes

The co-occurrence of QACs tolerance genes with other antimicrobial resistance genes (e.g., antibiotics, metals) in the same genetic contexts and the similarity of these QACs tolerance genetic contexts between *Enterococcus* and other bacterial taxa were evaluated.

Considering the few complete genomes included in the analysis for the different taxa, namely of *Enterococcus* spp., most QACs tolerance genes were found on plasmids of different sizes (*n* = 18/19; 2–129 kb) rather than in the chromosome (*n* = 1/19) (Fig. S1). For the most part, the genetic contexts of QACs tolerance genes studied presented high variability among the different bacterial taxa (Fig. S1-A-G), with exception of some isolates sharing few genes. This was the case for *E. faecium* C132 and *Staphylococcus aureus* CC1-1 or *Staphylococcus warneri* Ani-LG-025, sharing the *qacA/B* and beta-lactam resistance genes, or *E. faecalis* C32 and *Staphylococcus capitis* 15–101 or *Staphylococcus massiliensis* P3, sharing the *qacA/B* and copper tolerance genes (Fig. S1-A), among others. *Enterococcus* spp. from the clinical and environmental settings shared *qacZ* genetic contexts carrying *aac(6′)-Ie-aph(2′′)-Ia,* coding for aminoglycoside resistance, insertion sequences, and recombinases (Fig. S1-D). Also, *qrg* genetic contexts were shared between *E. faecalis* and *Streptococcus* spp.*,* mostly presenting insertion sequences, recombinases, and hypothetical proteins (Fig. S1-E). Finally, *E. faecalis* from different epidemiological backgrounds shared *bcrABC* genes, insertion sequences, genes coding for recombinases, hypothetical proteins, or bacteriocin associated (Fig. S1-F).

Regardless of the strains’ source, geographical region, or date of isolation, we observed that different genetic determinants for antibiotic resistance and metal tolerance were located adjacent to QACs tolerance genes. Of note, several of the genetic contexts analyzed (*n* = 32) in diverse bacteria, including *Enterococcus* spp., harbored genes conferring resistance to aminoglycosides, beta-lactams, macrolides, lincosamides, streptogramin B, mupirocin, bleomycin, tetracycline, chloramphenicol/florfenicol, trimethoprim, fosfomycin, and/or sulfonamide (Fig. S1-A,B,C,D,F,G). Metal tolerance genes, namely to copper, cadmium, zinc, or arsenic, were also detected within the vicinity of *qacA/B*, *qacC*, *qacJ*, *qrg*, and *bcrABC* genes (Fig. S1-A,B,C,E,F). Additionally, the genetic contexts analyzed were highly enriched in insertion sequences, recombinases, and replication associated proteins.

### QACs susceptibility assays of *E. faecalis* and *E. faecium*

The susceptibility of 105 *E. faecalis* and 105 *E. faecium* isolates (including two isolates carrying *qacZ*), with diverse epidemiological and clonal backgrounds (Table S2), to BC and DDAC was determined.

**Fig 2:**
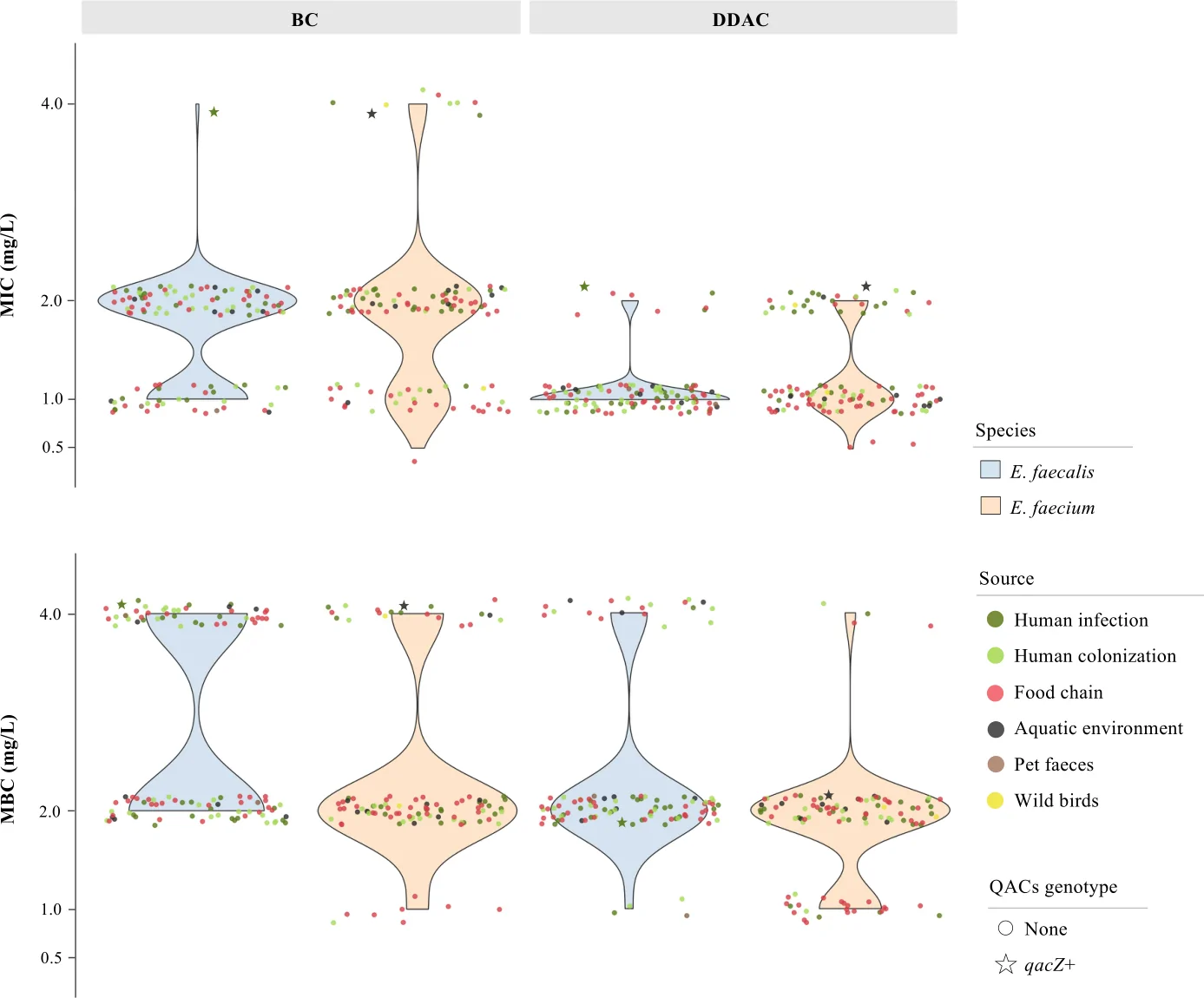
Benzalkonium chloride (BC) and didecyldimethylammonium chloride (DDAC) minimum inhibitory concentrations (MIC) and minimum bactericidal concentrations (MBC) distribution of *Enterococcus faecalis* (blue) and *Enterococcus faecium* (orange) isolates from diverse epidemiological backgrounds. MIC were established by broth microdilution in standard growth conditions [Mueller-Hinton broth (MHB); pH = 7.2; 37°C/20 h], followed by MBC determination, using the methodological approach proposed by the Clinical and Laboratory Standards Institute for antimicrobial susceptibility testing (33, 34). The source and QACs genotype of each isolate are indicated with different colors and shapes, respectively. For more isolates’ details see Table S2. Graphics were done in R, using the ggplot2 package v3.4.0 (35).

The MIC_BC_ in standard conditions were similar for the *E. faecalis* and *E. faecium* studied (MIC_50_ = 2 mg/L and MIC_90_ = 2 mg/L for both) (Fig. 2). The highest MIC_BC_ of 4 mg/L was observed in 1 *E. faecalis* and 9 *E. faecium* recovered from different sources, years, and clonal lineages. Of these, most were MDR (*n* = 8/10; resistant to three or more antibiotics from different families), and two of them harbored the gene *qacZ* (ST17 *E. faecium*-E241 and ST6 *E. faecalis*-V583). MBC_BC_ distributions for both species also showed similar MBC_50_ = 2 mg/L and MBC_90_ = 4 mg/L (Fig. 2). Likewise, the 43% (*n* = 45/105) of *E. faecalis* and 19% (*n* = 20/105) of *E. faecium* isolates showing the highest MBC_BC_ of 4 mg/L comprised, in both cases, isolates from different epidemiological and genetic backgrounds. On the contrary, *E. faecium* (*n* = 8) with the lowest MBC_BC_ of 1 mg/L were mostly from the food chain (*n* = 7/8), belonging to different STs and years. The value of MIC_BC_ and MBC_BC_ of the control strain *E. faecalis* ATCC 29212 varied between 1-2 mg/L and 2-4 mg/L, respectively.

Both species were susceptible to lower concentrations of DDAC compared to BC (*P* ≤ 0.01) (Fig. 2). For DDAC, the MIC_50_ was 1 mg/L for both species, and the MIC_90_ was 1 mg/L or 2 mg/L for *E. faecalis* and *E. faecium*, respectively (Fig. 2). Strains with an MIC_DDAC_ of 2 mg/L (*n* = 8 *E*. *faecalis* and *n* = 29 *E. faecium*) were diverse and comprised most of the *Enterococcus* with the highest MIC_BC_ (*n* = 7/10, including the two isolates carrying *qacZ*). Finally, the MBC_50_ was 2 mg/L for both species, and the MBC_90_ was 4 mg/L and 2 mg/L for *E. faecalis* and *E. faecium,* respectively. Again, isolates showing the highest MBC_DDAC_ of 4 mg/L or the lowest MBC_DDAC_ of 1 mg/L (18%, *n* = 19/105, and 4%, *n* = 4/105, of the *E. faecalis;* 4%, *n* = 4/105, and 23%, *n* = 24/105, of the *E. faecium* populations tested, respectively) were associated with different sources, years, and STs. The MIC_DDAC_ and MBC_DDAC_ of the control strain *E. faecalis* ATCC 29212 varied between 0.5-1 mg/L and 1-2 mg/L, respectively.

Considering all sources, *E. faecalis* seem to be more tolerant to bactericidal concentrations of the QACs tested (higher MBC) than *E. faecium* (*P* ≤ 0.01), although the latter presented a significantly higher MIC_DDAC_ (*P* ≤ 0.01) (Fig. 2). The MIC and MBC distributions of the isolates tested were analyzed separately by source and time span (5-year intervals) (Fig. S2), with the following significant differences among them: the MIC_DDAC_ and MIC_BC_ were higher for *E. faecium* recovered from human infections compared to those from the food chain (*P* ≤ 0.01), but a significant increasing trend in the MIC_BC_ over the years was detected in *E. faecium* isolates from the food chain (*P* ≤ 0.01) (Fig. S2). QACs susceptibility among MDR and non-MDR *E. faecalis* and *E. faecium* was similar (*P* > 0.01) (Fig. S3). BC and DDAC MIC and MBC values for vancomycin- or linezolid-resistant isolates varied within the ranges described for the whole population (Fig. S3).

**Fig 3:**
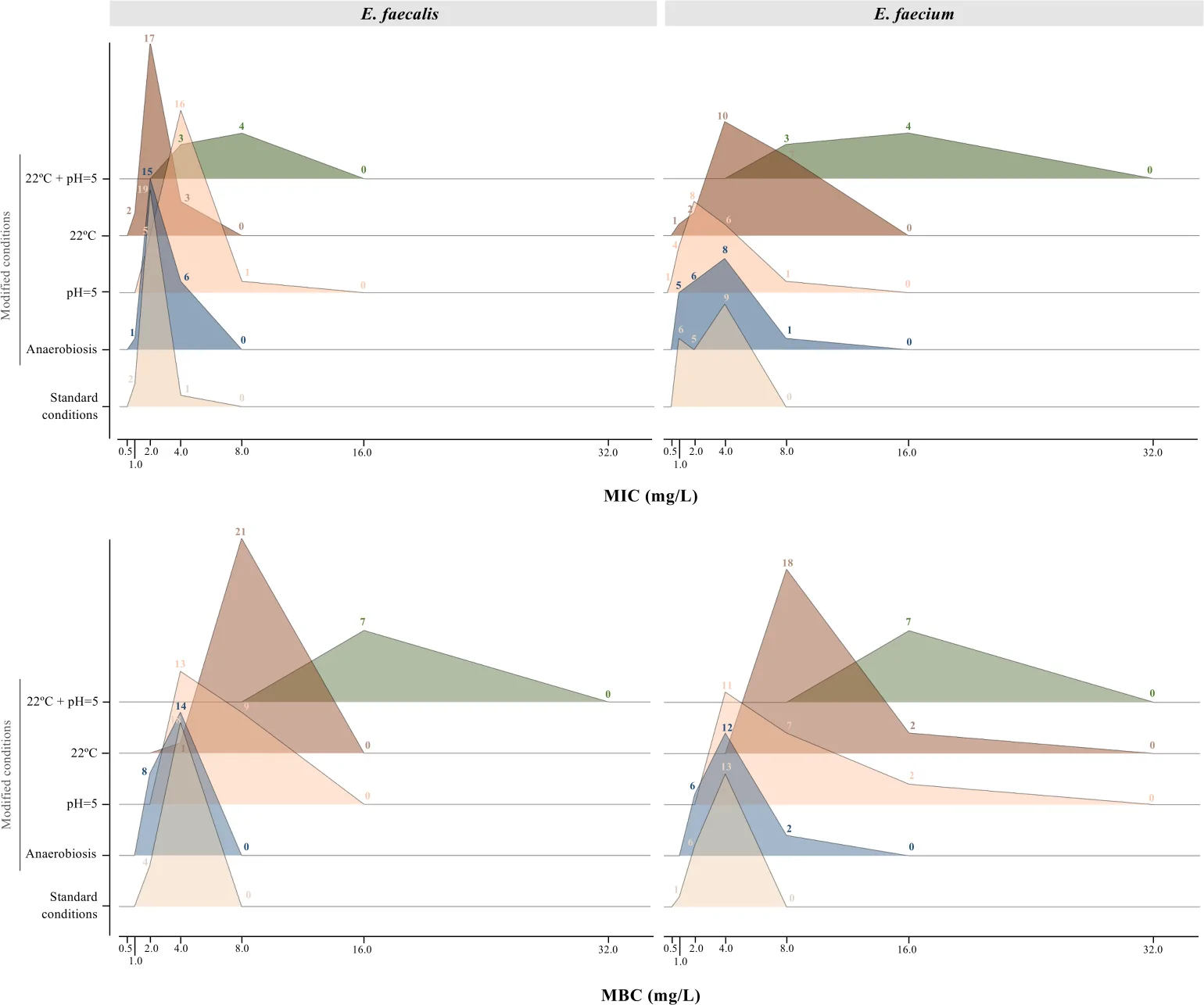
Ridgeline chart depicting the distribution of benzalkonium chloride minimum inhibitory concentrations (MIC) and minimum bactericidal concentrations (MBC) determined in standard or modified conditions. The non-standard conditions tested were anaerobiosis, room temperature (22°C), or mildly acidic pH (pH = 5) for 22 *Enterococcus faecalis* and 20 *Enterococcus faecium* isolates and a combination of 22°C and pH = 5 for 7 strains of each species. For more isolates’ details see Table S3. Graphics were built using the ggridges R package v0.5.4 (36).

### Different growth conditions occurring in the environment affect *Enterococcus* spp. susceptibility to QACs

To assess the activity of QACs on *Enterococcus* growth or survival under diverse environmental conditions that mimic real contexts, MIC_BC_ and MBC_BC_ were determined in anaerobic conditions (e.g., occurring in sewage) (37, 38), room temperature (22°C; representing abiotic surfaces in healthcare or agri-food sectors) (39), and mildly acidic pH (pH = 5; resembling human skin and fresh fruits/vegetables contaminated with QACs residues) (40 – 42). BC was the QAC chosen for the susceptibility assays with modified conditions, as both species demonstrated higher tolerance and phenotype variability (wider MIC and MBC ranges) to this compound compared to DDAC under standard conditions. MIC_BC_ and MBC_BC_ distributions for the modified conditions studied are shown in Fig. 3.

The analysis revealed similar results between aerobic (standard) and anaerobic conditions (*P* > 0.01), but increased tolerance to BC at room temperature (22°C) and/or mildly acidic pH (pH = 5) conditions for *E. faecalis* and *E. faecium* strains (*P* ≤ 0.01) (Fig. 3). In anaerobic conditions, MIC_BC_ or MBC_BC_ increased by no more than twofold in a few strains (six *E. faecalis* and six *E. faecium*) belonging to diverse sources, dates, and STs. The MBC_BC_ of *qacZ* + ST17 *E*. *faecium* E241 increased from 4 to 8 mg/L, outside the range obtained in standard conditions. Also, the MIC_BC_ and MBC_BC_ increased from 4 to 8 mg/L in a human *E. faecium* strain (ST412), recovered from a clinical infection in 2011, that did not contain any of the known QACs tolerance genes.

Contrastingly, four to eightfold MIC_BC_ and MBC_BC_ increases were observed at 22°C and/or pH = 5 conditions in diverse strains of both species. When isolates were tested at pH = 5, the MIC_BC_ increased significantly for *E. faecalis* (*P* ≤ 0.01) but not for *E. faecium* (*P* > 0.01), although MBC_BC_ were higher for both (*P* ≤ 0.01) (Fig. 3). The MIC_BC_ of one *E. faecalis* increased fourfold (from 1 to 4 mg/L; urban wastewater treatment plant from Tunisia, ST23, 2014) and the MIC_BC_ of the *qacZ + E. faecalis* V583 increased from 4 to 8 mg/L. The highest MBC_BC_ obtained among the investigated *E. faecalis* strains was also 8 mg/L, observed in nine strains exhibiting a twofold (*n* = 8; from several sources and STs) or fourfold rise (*n* = 1; poultry carcass, ST843, 2018). In the case of *E. faecium* isolates, the highest MBC_BC_ at pH = 5 was 16 mg/L, corresponding to *qacZ + E. faecium* E241 and one strain isolated from the faeces of a long-term care facility patient in 2016 (ST262). It is noteworthy that these strains exhibited an MBC_BC_ of 4 mg/L under standard conditions.

An increased tolerance to BC was more pronounced at 22°C than at pH = 5 for *E. faecium*, reflected both in MIC_BC_ (*n* = 3 isolates with fourfold and *n* = 2 isolates with eightfold increases at 22°C, compared to standard conditions) and MBC_BC_ (*n* = 6 isolates with fourfold and *n* = 2 isolates with eightfold increases at 22°C) values. The highest MBC_BC_ of 16 mg/L was detected in two food chain isolates (ready-to-eat salad and poultry carcass, ST12 and ST352, 2010–2019), while the remaining strains had an MBC_BC_ of 8 mg/L (*n* = 18). Among *E. faecalis,* in all but one of the isolates, the MBC_BC_ increased to 8 mg/L [following twofold (*n* = 17) or fourfold (*n* = 4) MBC_BC_ increases] at 22°C, whereas the MIC_BC_ did not change (*P* > 0.01) for most strains (*n* = 19).

The effect of the combination of the test conditions (growth at 22°C and pH = 5) was also studied, excluding anaerobiosis since it did not significantly influence QACs susceptibility. Indeed, higher MIC_BC_ or MBC_BC_ were observed for both species under 22°C and pH = 5 stress than when each condition was tested separately (*P* ≤ 0.01). MBC_BC_ increased from 1 to 4 mg/L (standard conditions) to 8 mg/L at 22°C (93%; *n* = 21/22 *E. faecalis* and *n* = 18/20 *E. faecium*) and 16 mg/L (100%; *n* = 7/7 *E. faecalis* and *n* = 7/7 *E. faecium*) for the combination of 22°C and pH = 5 for most isolates, regardless of the species, epidemiological or genetic background. MIC_BC_ also reached the highest values of 16 mg/L in four *E. faecium* strains from human infection and colonization, hospital sewage (*E. faecium* ST17 carrying *qacZ*), and food chain (2002–2020), and of 8 mg/L in four *E. faecalis* from human infection (*n* = 2, including an ST6 strain carrying *qacZ*), food chain, and environmental origins (1987–2019).

**Fig 4:**
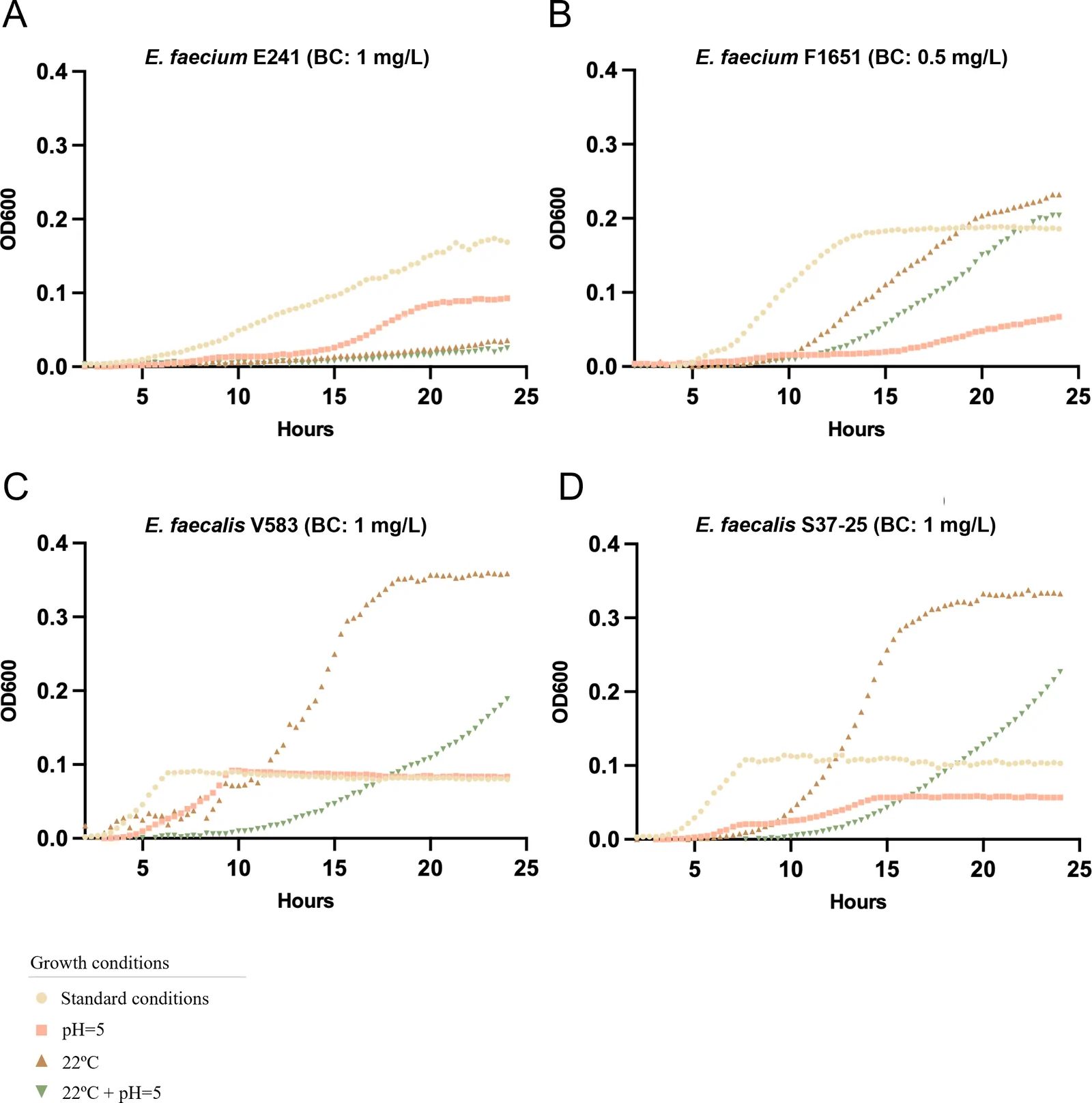
Growth kinetics of two *Enterococcus faecium* and two *Enterococcus faecalis* in the presence of subinhibitory concentrations of benzalkonium chloride [1 mg/L for *E. faecium* E241 with *qacZ* (**A**), *E. faecalis* V583 with *qacZ* (**C**), and *E. faecalis* S37-25 without *qacZ* (**D**) and 0.5 mg/L for *E. faecium* F1651 without *qacZ* (**B**)] in standard (light-yellow circles) or modified conditions. The non-standard conditions tested were 22°C (light pink squares), mildly acidic pH = 5 (brown triangles) and a combination of 22°C and pH = 5 (inverted green triangles). For more isolates’ details see Table S3. Growth curves were determined using a Biotek Synergy HT plate reader (Marshall Scientific), with absorbance at 600 nm (A600) recorded every 20 minutes for 24 hours. Graphics were built using the Prism software v9.0 (GraphPad Software; www.graphpad.com).

Two *E. faecalis* (V583 with *qacZ*; S37-25 without *qacZ*) and two *E. faecium* (E241 with *qacZ*; F1651 without *qacZ*) were included in kinetic assays, showing changes in their growth dynamics under the modified conditions studied compared with the standard ones (Fig. S4 and S5). Such modifications in the growth curves were strain specific, with *E. faecalis* isolates showing more similarities among each other compared to the *E. faecium* isolates. This was observed in the kinetic assays conducted both with subinhibitory concentrations of BC (0.5 mg/L for *E. faecium* F1651 and 1 mg/L for *E. faecium* E241, *E. faecalis* V583, and S37-25) as well as without BC (Fig. 4; Fig. S4 and S5). Comparing the growth kinetics of each strain under BC plus modified conditions with the standard ones (Fig. 4), different bacterial adaptations were observed, including extended lag phases and, in several cases, slower exponential growth, resulting in a delayed entry into the stationary phase. Also, for the *E. faecium* F1651 and the two *E. faecalis*, their growth with BC plus 22°C or 22°C + pH = 5 surpassed that occurring in standard conditions during the time of the assay, which may suggest that these modified conditions are better for bacterial multiplication under BC stress.

Specifically, at pH = 5 or 22°C, we described earlier that both *E. faecium* and *E. faecalis* increased their MBC_BC_ (two to eightfold) compared to standard conditions, which may be explained by the longer exponential growth phase in such modified conditions (Fig. 4). Additionally, the highest MBC_BC_ increase observed for *E. faecium* (eightfold) may be related to their exponential growth for even longer periods compared to *E. faecalis,* which enter much earlier into the stationary phase (<20 hours) (Fig. 4). Of note, for the combination of 22°C + pH = 5 conditions, growth kinetics for all *E. faecium* and *E. faecalis* strains included in the analysis have shown that they were still in the exponential multiplication phase after 24 hours. This may explain the higher MBC_BC_ observed for both species under the combination of 22°C and pH = 5 stress than for any other growth condition tested, with all isolates reaching the highest MBC_BC_ of 16 mg/L. No clear differences were detected in isolates carrying the *qacZ* gene (*E. faecium* E241; *E. faecalis* V583) or not (*E. faecium* F1651; *E. faecalis* S37-25) in any condition tested.

### Pre-exposure to BC does not increase *E. faecalis* and *E. faecium* tolerance to the biocide

In order to assess if a pre-exposure to QACs would increase QACs-tolerant phenotypes, we exposed seven *E. faecalis* and seven *E. faecium* strains to subinhibitory concentrations of BC (0.5× MIC_BC_) at different contact times (3, 5, and 10 minutes), but no change in their susceptibility to BC was observed (*P* > 0.01). This was also the case for the *E. faecium* and *E. faecalis* isolates harboring *qacZ*.

## DISCUSSION

This study highlights the increased tolerance of *Enterococcus* spp. to QACs under different environmental stresses, although QACs typical in-use concentrations remain effective in all conditions tested. Furthermore, it shows that *Enterococcus* spp. share known QACs tolerance genes with diverse bacterial taxa within the same ecosystems, despite the low content of QACs tolerance genes among enterococci. These findings show the importance of understanding the complex dynamics of QACs tolerance in microbial communities and provide new opportunities for further exploration in this field.

Despite the increased use of QACs in recent years (4, 5), *E. faecalis* and *E. faecium* from various epidemiological backgrounds remain susceptible to QACs concentrations lower than those recommended for disinfection or preservation, with MIC and MBC distributions for BC and DDAC being consistent with previous reports (10, 20, 22, 27, 43). The observed similar QACs phenotypes in isolates with and without *qacZ* gene, along with supporting published data (20, 22), suggest that QACs tolerance genes may not have a significant impact on the survival of *Enterococcus* spp. under QACs exposure. It is also possible that the standard susceptibility determination methods did not accurately replicate the necessary conditions for the expression of QACs tolerance genotypes. The apparent lack of selective advantage conferred by QACs tolerance genes in *Enterococcus* seems to be also corroborated by their low prevalence among *Enterococcus* isolates, which could be explained by rare horizontal transfer events, lack of stability of the acquired genes or genetic contexts due to, for example, high fitness costs (11). Accordingly, literature mostly supports that *Enterococcus* from diverse sources do not seem to be relevant reservoirs of known QACs tolerance genes (20 – 22, 43), with few exceptions (24, 25). In addition, we found that pre-exposure to BC did not induce a higher tolerance to this biocide in isolates carrying *qacZ*, which may indicate that the BC concentration and exposure times tested were not able to induce the gene expression of the efflux pump encoded by *qacZ*, that increased expression levels of *qacZ* did not lead to a detectable increase in *Enterococcus* tolerance to BC, or that other gene expression regulatory systems or environmental inductors are needed. In a few studies, bacterial adaptation and higher tolerance to QACs have been observed following repeated exposure to sublethal/increasing concentrations of QACs (44, 45), but QACs tolerance genes were either not considered or detected, making it difficult to draw conclusions about their role on the increased phenotypes.

The main source and bacterial species harboring identical QACs proteins that were found in *Enterococcus* spp. might reflect the respective microbial communities and settings in which specific QACs tolerance genes circulate. For example, the genes *qac* and *qrg*, identified mostly in human *Enterococcus* were, respectively, predominant in *Staphylococcus* and *Streptococcus* isolates mainly associated with human colonization and infection (46 – 48), whereas *bcrABC* and *oqxAB,* mostly found in *Enterococcus* spp. recovered from the food chain, were largely detected in bacterial species often occurring as food chain pathogens or hygiene indicators, such as *Listeria* spp. and *E. coli* (49 – 51). The genetic contexts of QACs tolerance genes studied were very diverse in *Enterococcus* and other taxa, probably as a result of a high number of recombination events, as suggested by the abundance of insertion sequences and recombinases detected. They were often co-located with genes coding for resistance or tolerance to other antimicrobial agents, such as antibiotics or metals, coexisting as selective agents in many ecosystems (52 – 54), and highlighting the possibility of co- and cross-selection events.

However, phenotypes of reduced susceptibility to QACs have not only been attributed to the acquisition of particular genes. It has been suggested that environmental conditions may contribute to bacterial cellular adaptations protective against the action of biocides (29). Here, when testing bacterial susceptibility to QACs under conditions such as lower temperatures than ideal for growth (e.g., room temperature, as found on many abiotic surfaces) (39) and/or mildly acidic pH (e.g., on human skin or on fruits and vegetables) (40 – 42), *Enterococcus* tolerance to BC significantly increased and growth kinetics changed, regardless of whether they contained or not known QACs tolerance genes. Environmental stresses can cause cellular modifications in both Gram-positive and Gram-negative bacteria (e.g., changes in membrane composition and fluidity, cellular metabolism), with implications on the activity of cationic compounds, such as QACs (29, 55 – 57). Although the effects of these stresses on *Enterococcus* spp. susceptibility to QACs are still rather unexplored, in other Bacillota, such as *Listeria* spp. or *S. aureus*, studies have shown that adaptation to acid pH, lower temperature, and anaerobiosis resulted in strains more tolerant to different QACs (58 – 61). Also, similar findings have been reported for Gram-negative bacteria (55, 62).

While the more tolerant phenotypes detected in this study at pH = 5 and/or 22°C were found to be below the in-use QACs concentrations, the potential implications of environmental stress on biocide efficacy emphasize the importance of conducting susceptibility tests under conditions mimicking those occurring in settings where *Enterococcus* are exposed to QACs. Also, QACs concentrations within or below the MIC and MBC ranges obtained in this study have been detected in wastewater and surface waters (less than 0.001 to 6 mg/L) as well as in various types of food, including fruits and nuts, meat, or dairy products (up to 14.4 mg/kg) (6, 7, 42), and might promote the selection and persistence of particular strains, possibly increased by environmental factors. Moreover, exposure to sublethal concentrations of disinfectants, including QACs, has been associated with an increased horizontal gene transfer via conjugation by upregulating the SOS response, enhancing the membrane permeability and production of reactive oxygen species (63 – 65).

In conclusion, this study provides a comprehensive phenotypic and genomic analysis of the *E. faecalis* and *E. faecium* tolerance to QACs. Although *Enterococcus* do not seem to be significant hosts of QACs tolerance genes, they can adapt to modified growth conditions occurring in the environment toward more tolerant phenotypes. Further studies, including whole transcriptome analyses, are needed to understand the expression of known QACs tolerance genes and the cellular mechanisms influencing *Enterococcus* adaptation to QACs. Continuous surveillance, namely through longitudinal metadata studies considering the influence of environmental interventions and local physicochemical factors on the dynamics of the microbiota composition in different settings, will also be crucial to assess the selection of tolerant *Enterococcus* populations exposed to varying concentrations of QACs. Such research can offer valuable insights for One Health intervention strategies aimed at preventing future biocide inefficacy, with significant implications for Public Health.

## MATERIALS AND METHODS

### Epidemiological background of the bacterial isolates studied

A collection of 105 *E. faecium* and 105 *E. faecalis* isolates, representative of different geographical regions, sources, time spans, and genomic backgrounds, was selected for this study (Table S2). They were recovered in previous studies from human infections (*n* = 53), human colonization (*n* = 47), food chain (food-animal production settings, meat of animal origin, and other food products) (*n* = 89), pets (*n* = 2), wild birds (*n* = 2), and aquatic environment (*n* = 17) samples, in diverse regions (Portugal, Tunisia, Angola, Brazil, Spain, Germany, Canada, United States) and time spans (1996–2020) (Table S2) (66 – 71). Among them, 73% (*n* = 77/105) of *E. faecium* and 43% (*n* = 45/105) of *E. faecalis* were classified as MDR (resistance to three or more antibiotics from different families), 34% (*n* = 36/105) and 10% (*n* = 11/105) as resistant to vancomycin and 5% (*n* = 5/105) and 4% (*n* = 4/105) to linezolid, respectively (Table S2) (66 – 71). Clonal relationship was established by multilocus sequence typing (MLST; sequence-type-ST) (72 – 74) and core genome MLST (cgMLST; Complex Type-CT; Ridom SeqSphere+, version 8.2.0; https://www.ridom.de/seqsphere) (75, 76).

### Screening of QACs tolerance genes in *Enterococcus* spp. isolates and public genomes of diverse bacterial taxa

Several *qac* genes (*qacA/B, qacC, qacG, qacJ, qacZ*), the *qrg*, *bcrABC,* and the *oqxAB* genes (GenBank accession no. HE579074.1, GQ900434.1, Y16944.1, NG_048046.1, KM083808.1, HQ663849.2, NZ_CP011399.1, KT716391.1, respectively), coding for efflux pumps, were searched among the isolates included in the phenotypic assays. For the *E. faecium* (*n* = 61) and *E. faecalis* (*n* = 101) isolates sequenced in previous studies (67, 70, 77, 78), the genetic screening was performed using the MyDBfinder tool available at the Center for Genomic Epidemiology (www.genomicepidemiology.org). In the remaining isolates, the presence of genes associated with tolerance to QACs was identified by PCR, using primers and conditions described previously (21, 23, 79, 80).

Additionally, the frequency and distribution of *Enterococcus* strains with QACs tolerance genes were also assessed among *Enterococcus* genomes (*n* = 22,428, until 28 April 2022; https://www.ncbi.nlm.nih.gov/datasets/genome/?taxon=1350) and proteins from other collections available at the NCBI database. Amino acid sequences with ≥90% of query-cover and ≥97% identity were considered. The source of isolates carrying QACs tolerance genes was retrieved from the NCBI and BV-BRC (Bacterial and Viral Bioinformatics Resource Center) databases and their clonality determined at the PubMLST website (72 – 74).

Also, QACs tolerance proteins variants identified in *Enterococcus* were searched in other NCBI published bacterial taxa genomes (*n* = 1,162,672, until 28 April 2022) and proteins, in order to infer about the genetic flow of QACs tolerance genes. This was performed using the blastp tool (81), by looking at the 100% identical amino acid sequences (coverage and identity) of each of the proteins found in *Enterococcus* strains. The source of the isolates of all taxa carrying such QACs tolerance proteins variants was retrieved from the NCBI Biosample database (http://www.ncbi.nlm.nih.gov/biosample/), when available.

### Comparative analysis of the genetic contexts of QACs tolerance genes

The synteny and nucleotide identity of the genetic contexts of QACs tolerance genes among *Enterococcus* and other bacterial taxa genomes were analyzed using the BLASTN option of Easyfig v2.2.2 (82) and based on NCBI genome annotations. To assess the genetic context of each QACs tolerance gene, *Enterococcus* strains and one representative species from other bacterial taxa from several sources and time spans were selected for comparison. The criteria used to select the boundaries of the analyzed sequences were the limit of the contigs in which the QACs tolerance genes were located or the presence of genetic elements of interest (e.g., antibiotic resistance, biocides or virulence genes, mobile genetic elements).

### QACs susceptibility assays in standard conditions

The MIC_BC_ (BC: CAS 68391–01-5, VWR) and MIC_DDAC_ (DDAC: CAS 7173–51-5, Sigma Aldrich) of the 210 *Enterococcus* isolates were established by broth microdilution, using the methodological approach proposed by the Clinical and Laboratory Standards Institute (CLSI) for antimicrobial susceptibility testing [Mueller-Hinton broth (MHB); pH = 7.2; 37°C/20 hours] (33). A 96-well microtiter plate containing serial twofold dilutions of the disinfectant (concentration range of 0.125 to 128 mg/L) was used to assess the susceptibility of bacterial suspensions in log-phase growth (adjusted to reach a final inoculum of 5 × 10^5^ CFU/mL in each well) at 37°C for 20 hours. Microdilution panels were freshly prepared before each assay. The first concentration of QAC without visible growth was considered the MIC (33).

To determine the MBC_BC_ and MBC_DDAC_, 10 µL of each well without visible growth from the 96-well MIC plate was incubated onto brain heart infusion (BHI) agar plates at 37°C for 24 hours, as defined by the CLSI (34). The MBC was defined as the lowest QAC concentration for which the number of colonies was equal or less than the rejection value defined by CLSI guidelines, based on the final inoculum of each well confirmed by actual count (34). Each experiment was repeated three to six times, and the MIC/MBC values corresponded to the mean of the determinations. The *E. faecalis* ATCC 29212 strain (without any known QACs tolerance genes) was included as control to guarantee the reproducibility of all assays. The MIC_50_ and the MIC_90_ (minimum concentration of an antimicrobial agent that inhibits the growth of 50% or 90% of the bacterial population, respectively) as well as the MBC_50_ and the MBC_90_ (lowest concentration of an antimicrobial agent that is required to kill 50% or 90% of the bacterial population, respectively) were determined.

### QACs susceptibility in modified environmental conditions

QACs assays performed in modified environmental conditions (anaerobiosis, pH = 5, 22°C, or pH = 5 + 22°C) were performed for 20 *E. faecium* and 22 *E. faecalis* (Table S3). The bacterial strains selected were representative of the MIC and MBC distribution ranges in standard conditions, different geographical regions, sources, time spans, antimicrobial resistance profiles, and genomic backgrounds of the initial collections. Modified conditions were initially tested separately (anaerobiosis, pH = 5, 22°C), and subsequently, MIC_BC_ and MBC_BC_ were determined with cross-environmental conditions (pH = 5 + 22°C) for seven *E. faecium* and seven *E. faecalis* (Table S3), based on the results obtained from individual assays. From these 14 isolates, two *E. faecium* isolates (E241 with *qacZ* from hospital sewage and F1651 without *qacZ* from poultry meat, belonging to ST17 and ST12, respectively) and two *E. faecalis* isolates (V583 with *qacZ* from clinical origin and S37-25 without *qacZ* from a ready-to-eat salad, identified as ST6 and ST309) were selected for kinetic assays. These assays, which provide more quantitative information compared to the endpoint measurements used in MIC and MBC determinations, were performed under different growth conditions including standard conditions (37°C; pH = 7.2), pH = 5, 22°C, and pH = 5 + 22°C. BC concentrations tested corresponded to the 0.5× MIC_BC_ of the lowest MIC_BC_ obtained in the three replicates in standard conditions for each strain. Growth curves were determined using a Biotek Synergy HT plate reader (Marshall Scientific), with absorbance at 600 nm (A600) recorded every 20 minutes for 24 hours. Graphics were built using the Prism software v9.0 (GraphPad Software; www.graphpad.com).

### QACs susceptibility after bacteria pre-exposure to BC

The seven *E. faecalis* and seven *E. faecium* strains previously selected for cross-stress testing (including *qacZ* + ST17 *E*. *faecium* E241 and ST6 *E. faecalis* V583) (Table S3) were pre-exposed to BC. The protocol was designed based on the European standards EN 1276 (83) and EN 13727 (84) and a methodology previously described by Skive et al. (85). Briefly, MHB bacterial suspensions in log-phase of growth were split into two glass tubes and exposed to either 0.5× MIC_BC_ or to only MHB as a control. The cultures were left in the presence of BC for 3, 5, or 10  minutes at room temperature to determine the effect of usually recommended disinfectant contact times (17, 86, 87). Following exposure, 1 mL of the suspension was added to 9 mL of neutralizer solution prepared according to EN 13727 (84) containing Tween 80 (30 g/L; CAS: 9005-65-6, Sigma Aldrich), sodium dodecyl sulfate (4 g/L; CAS: 151-21-3, VWR), and lecithin (3 g/L; MP Biomedicals). Cells were centrifuged at 2,500 × *g* for 10  minutes. Supernatant was removed and cells were resuspended in 3  mL of PBS (phosphate-buffered saline). Cells were centrifuged at 2,500 × *g* for 10  minutes, and supernatant was removed from the pelleted cells. The washed cells were resuspended in 2  mL of NaCl 0.9% and volume adjusted to reach a final inoculum of 5 × 10^5^ CFU/mL in each well. MIC_BC_ and MBC_BC_ were then determined as described above, in standard conditions, following the CLSI guidelines (33, 34).

The effectiveness and toxicity of the neutralizer were evaluated according to the protocol described by Vali et al. (88). First, neutralizer toxicity was tested by adding 1 mL of neutralizer to a bacterial suspension and left in contact for 5 minutes. Cells were serially diluted to reach a final inoculum of 5 × 10^5^ CFU/mL and incubated in Mueller-Hinton agar plates at 37°C for 24 hours. The number of CFU/mL was compared to a control with NaCl 0.9% replacing the neutralizer. To confirm BC was being quenched effectively by the neutralizer, a bacterial suspension was split into three glass tubes and, in each, was added a mixture containing 1 mL of biocide (at bactericidal concentration) and 9 mL of neutralizer, a mixture of 1 mL biocide (at bactericidal concentration) and 9 mL NaCl 0.9%, or 10 mL NaCl 0.9%. After 5 minutes of contact time, cells were diluted and incubated as previously, and the number of CFU/mL was compared. The results for the effectiveness and toxicity test of the neutralizer are shown in Table S4.

### Statistical analysis

The statistical significance of the differences between MIC and MBC distributions of isolates from the diverse sources, time spans, and with disparate antibiotic resistance profiles in standard conditions, as well as differences in MIC_BC_ and MBC_BC_ between standard and modified conditions, were assessed using the Wilcoxon Signed Rank and Mann-Whitney Wilcoxon tests (R Statistical Software, v4.1.2) (89). The statistical analysis of potential associations between QACs tolerance genes found in *Enterococcus* and particular bacterial taxa or sources was performed by Fisher’s exact test (Prism software, v9.0, GraphPad Software; www.graphpad.com). *P* values ≤0.01 were considered significant.

## Data Availability

Genome sequences used in this study have been deposited in GenBank in previous studies under the BioProject numbers PRJEB40976, PRJNA663240, PRJNA546230, and PRJNA800622.

## SUPPLEMENTAL MATERIAL

Supplemental material spectrum.02324-23-s0001.pdf Tables S1, S2, S3, and S4 and Figures S1, S2, S3, S4, and S5.

ASM does not own the copyrights to Supplemental Material that may be linked to, or accessed through, an article. The authors have granted ASM a non-exclusive, world-wide license to publish the Supplemental Material files. Please contact the corresponding author directly for reuse.
