# Supplementary material for: Unraveling Enterococcus susceptibility to quaternary ammonium compounds: genes, phenotypes, and the impact of environmental conditions
Source: Microbiol Spectr. 2023 Sep 22;11(5):e02324-23. doi: 10.1128/spectrum.02324-23 (PMC10581157; doi:10.1128/spectrum.02324-23)
Supplement: Supplemental material — Tables S1, S2, S3, and S4 and Figures S1, S2, S3, S4, and S5. [file spectrum.02324-23-s0001.pdf]

TABLE S1 – Epidemiological background of all *Enterococcus* spp. strains harboring *qac, qrg, bcrABC* and *oqxAB* genes published at the NCBI database (until the 28th of April 2021).

Gene(s)	Species	Isolate	ST	Date	Isolation Source	Country	GenBank Acc. Number
qacA/B	*E. faecalis*	C27	81	2019	Human infection	South Africa	NZ_JADBPE000000000
		C32	81	2019	Human infection	South Africa	NZ_JADBPP000000000
	E. faecium	C132	761	2019	Human infection	South Africa	NZ_JADBPD000000000
qacC	*E. faecalis*	SF28073 (EnGen0243)	2	2003	Human infection	USA	NZ_AJBC00000000
		4	2	2010	Human colonization	USA	DACBBL000000000
		19	2	2010	Human colonization	USA	DACAXD000000000
		352	2	2010	Human colonization	USA	DACBCW000000000
		1238	2	2011	Human colonization	USA	DACAYD000000000
		1750	2	2011	Human colonization	USA	DACBAN000000000
		31	2	2012	Human colonization	USA	DACAZT000000000
		3448	2	2012	Human colonization	USA	DACBDB000000000
		3447	2	2012	Human colonization	USA	DACBDD000000000
		3366	2	2012	Human colonization	USA	DACAYK000000000
		3367	2	2012	Human colonization	USA	DACAYL000000000
		ERV31	2	NA	Human	NA	NZ_ALZN00000000
		ERV37	2	NA	Human	NA	NZ_ALZO00000000
		ERV129	2	NA	Human colonization	NA	NZ_ALZL00000000
		TX0104	2	NA	Human infection	NA	NZ_ACGL00000000
		BCW_3742	2	NA	NA	NA	DACKBI000000000
		C39	81	2019	Human infection	South Africa	NZ_JADBPK000000000
		Merz151 (EnGen0358)	104	2002	Human infection	USA	NZ_ASDS00000000
		VRE33500	254	2018	Human colonization	USA	NZ_JAEIZP000000000
		VRE33417	891	2018	Human colonization	USA	NZ_JAEIUF000000000
		VRE33393	891	2018	Human colonization	USA	NZ_JAEIVA000000000
		VRE33217	891	2018	Human colonization	USA	NZ_JAEIVU000000000
		VRE34953	891	2019	Human infection	USA	NZ_JAEJIO000000000
		VRE32555	891	2017	Human infection	USA	NZ_JAEJYH000000000
		VRE32929	891	2017	Human colonization	USA	DACBVV000000000
		VRE33136	891	2018	Human colonization	USA	NZ_JAEKAL000000000
		VRE33093	891	2018	Human colonization	USA	DACBWK000000000
		72	778	2014	Human colonization	USA	DACAXQ000000000
		ERV81	New	NA	Human	NA	NZ_ALZW00000000
	E. faecium	V138	80	2013	Human	Denmark	DACJDL000000000
		V37	80	2013	Human	Denmark	DACJFD000000000
		V63	117	2013	Human	Denmark	DADXYD000000000
		V1666	203	2016	Human colonization	Denmark	DAFCJN000000000
		V1668	203	2016	Human colonization	Denmark	DAFCJS000000000
		V1418	1297	2016	Human colonization	Denmark	DADQWF000000000
		E41209_2017	NA	2017	NA	Australia	DACECH000000000
qacG	*E. faecium*	8D1-48	1753	2018	Soil	South Africa	NZ_JAAMSA000000000
qacJ	*E. faecalis*	C30	81	2019	Human infection	South Africa	NZ_JADBPF000000000
qacZ	*E. faecalis*	V583	6	1987	Human infection	USA	NC_004668
		V587 (EnGen0242)	6	1987	Human infection	USA	NZ_AJBB00000000
		NCTC 12203	6	1987	Human infection	UK	NZ_UGIK00000000
		ATCC 51299	6	1993	Human infection	USA	NZ_JSES00000000
		ARL04-446	6	2004	Human infection	New Zealand	NZ_QPTF00000000
		UW4687	6	2004	Human colonization	Germany	DACBDW000000000
		UW7354	6	2008	Human	Portugal	DACBIG000000000
		BSAC-ec1762	6	2008	Human infection	UK	DACBPU000000000
		BSEF-36	6	2014	Human infection	USA	NZ_QAWN00000000
		BSEF-39	6	2014	Human infection	USA	NZ_QAWM00000000
		BSEF-42	6	2014	Human infection	USA	NZ_QAWL00000000
		UAA946 (EnGen0253)	26	1996	NA	USA	NZ_AIZQ00000000
		UAA948 (EnGen0232)	26	1996	NA	USA	NZ_AIZS00000000
		NCTC 12201	28	1986	Human infection	UK	NZ_UGIZ00000000
		BSAC-ec292	28	2002	Human infection	UK	DACBPJ000000000
		BSAC-ec1263	28	2006	Human infection	UK	DACBSK000000000
		CLB21560	28	2017	Human infection	USA	NZ_CP019512
		HC_NS0057	525	2015	Human	Canada	JABRHT000000000
		HC_NS0822	525	2015	Human	Canada	JABRLU000000000
		BSEF-44	New	2014	Human infection	USA	NZ_QAWK00000000

1 - Genes found among published *Enterococcus* spp. proteins and not complete genomes.

* - Strain from our collection.

Abbreviations: NA, not available; UK, United Kingdom; USA, United States of America.

TABLE S1 (continued) – Epidemiological background of all *Enterococcus* spp. strains harboring *qac, qrg, bcrABC* and *oqxAB* genes published at the NCBI database (until the 28th of April 2021).

Gene(s)	Species	Isolate	ST	Date	Isolation Source	Country	GenBank Acc. Number
qacZ	*E. faecium*	E241*	17	2002	Hospital sewage	Portugal	KM083808
		E7	18	2009	Human infection	USA	DACEKJ000000000
		32	18	2010	Human infection	Russia	NZ_PDFP00000000
		E290	18	2013	Human infection	USA	DACEMY000000000
		E301	18	2013	Human infection	USA	DACEKV000000000
		VRE33148	18	2018	Human colonization	USA	JADUPV000000000
		E178	282	2009	Human infection	USA	DACEIS000000000
		E438	282	2009	Human infection	USA	DACEMD000000000
		E440	282	2009	Human infection	USA	DACEOU000000000
		VRE34923	282	2019	Human colonization	USA	NZ_JADRDG000000000
		E180	736	2010	Human infection	USA	DACZFN000000000
		E0369	872	2015	Human	India	DADMXL000000000
		ISMMS_VRE_1	2272	2011	Human infection	USA	NZ_CP012430
qrg	*E. faecalis*	DVT_1043	6	2020	Human infection	USA	DAEEVS000000000
bcrABC	*E. faecalis*	3983	6	2013	Human colonization	USA	DACAYW000000000
		VRE32801	6	2017	Human colonization	USA	JAEKAV000000000
		G138E	21	2016	Beef plant	Canada	VHRY00000000
		FSIS11816003	22	2018	Swine	USA	AAXEIS000000000
		EF72PII	25	NA	Human infection	USA	DACAME000000000
		703-EFLS	40	2012-2013	Human infection	USA	JUWK00000000
		G149	40	2016	Beef plant	Canada	VHRX00000000
		FSIS12029501	40	2020	Catfish	USA	AAXDJB000000000
		BSAC-ec2240	73	2009	Human infection	UK	DACBSF000000000
		H44	76	2015	Beef carcass	Canada	VHRM00000000
		G119	76	2015	Beef processing facility	Canada	JAGLAU000000000
		G90	84	2015	Beef processing facility	Canada	JAGLAW000000000
		FSIS12142132	84	2020	Catfish	USA	ABCEYE000000000
		4928STDY7071666	97	2018	Human colonization	UK	CABGVE000000000
		263EA1	126	NA	Chicken meat	USA	LEPN00000000
		4928STDY7071628	133	2018	Human colonization	UK	CABGUD000000000
		1726	158	2011	Human colonization	USA	DACBAR000000000
		2015	158	2011	Human colonization	USA	DACBCG000000000
		3016	158	2011	Human colonization	USA	DACAZS000000000
		CVM N54595	192	2014	Pork chop	USA	PUAC00000000
		R49	192	2015	Retail beef	Canada	VHRC00000000
		CVM N53420	228	2014	Pork chop	USA	PTYL00000000
		R48	228	2015	Beef processing facility	Canada	JAGLBN000000000
		W100	260	2015	Beef carcass	Canada	VHQU00000000
		R37	260	2015	Retail beef	Canada	VHRE00000000
		R52	260	2015	Retail beef	Canada	VHQY00000000
		R53	260	2016	Retail beef	Canada	VHQX00000000
		CVM N55185	273	2014	Pork chop	USA	PUAM00000000
		CVM N52729	282	2014	Pork chop	USA	PTXY00000000
		4928STDY7071622	290	2018	Human colonization	UK	CABGTR000000000
		G81	624	2015	Beef plant	Canada	VHRU00000000
		EC1251	648	2009	Human infection	UK	DACBSY000000000
		W84	949	2015	Beef carcass	Canada	VHQP00000000
		R5E	New	2014	Retail beef	Canada	VHQW00000000
		G127E	New	2015	Beef plant	Canada	VHRZ00000000
		DVT_872	New	2019	Human infection	USA	DAEEVX000000000
		UBA9870	NA	NA	NA	NA	DQAR00000000
	E. faecium	42EA1	1950	NA	Chicken meat	USA	NZ_LERK00000000
	E. lactis	CVM N54519		2014	Ground beef	USA	NZ_PTZP00000000
oqxAB 1	*E. faecalis*	3EF	NA	2010-2012	Pig faeces	China	KT716391
		4EF	NA	2010-2012	Pig faeces	China	KT716392
		5EF	NA	2010-2012	Pig faeces	China	KT727030

1 - Genes found among published *Enterococcus* spp. proteins and not complete genomes.
* - Strain from our collection.
Abbreviations: NA, not available; UK, United Kingdom; USA, United States of America.

A

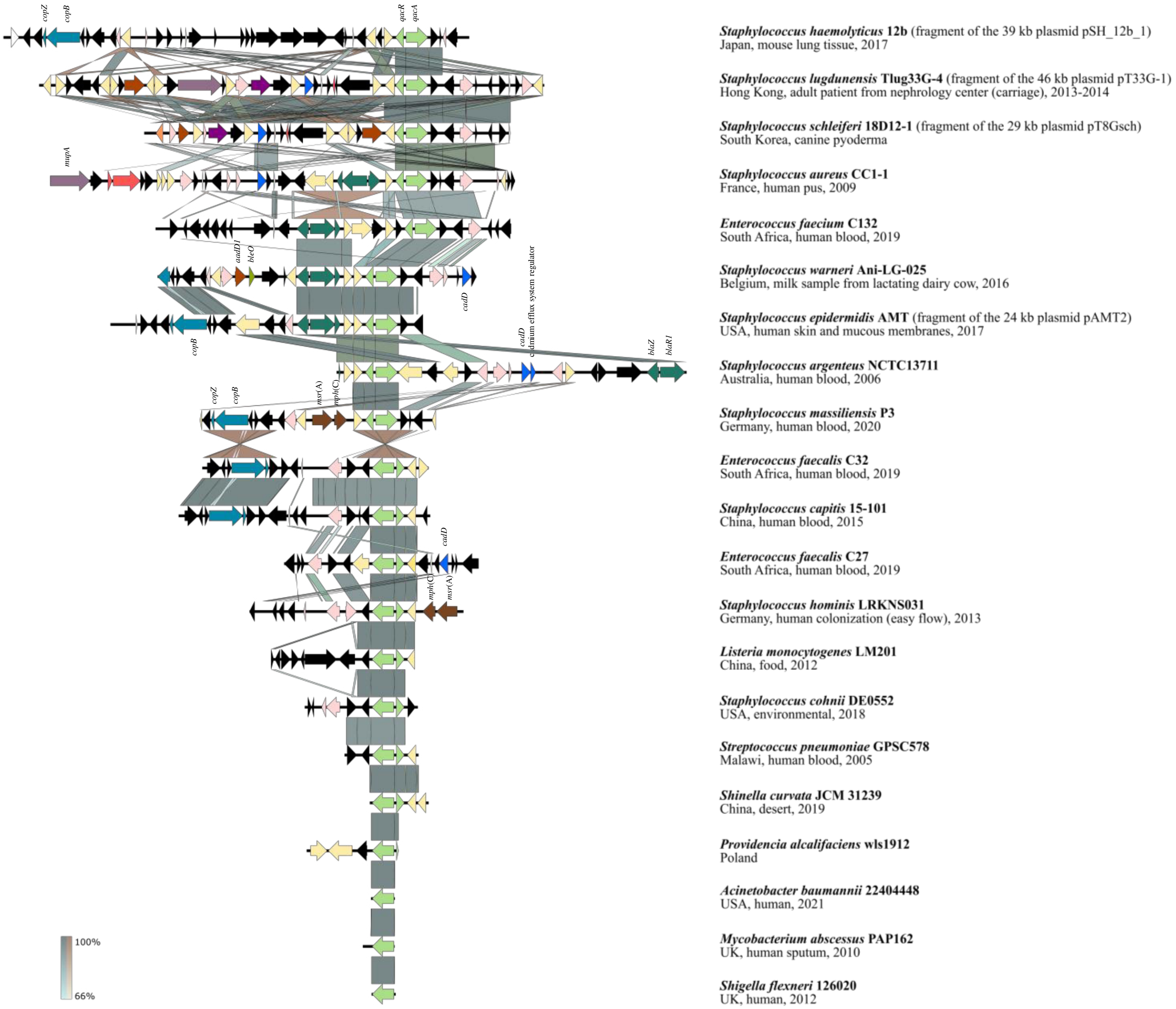

copZ
copB
qacR
qacA
mupA
aadD1
bleO
cadD
cadmium efflux system regulator
copB
cadD
blaZ
blaR1
copZ
copB
msr(A)
mph(C)
cadD
mph(C)
msr(A)
100%
66%
***Staphylococcus haemolyticus* 12b** (fragment of the 39 kb plasmid pSH_12b_1)
Japan, mouse lung tissue, 2017
***Staphylococcus lugdunensis* Tlug33G-4** (fragment of the 46 kb plasmid pT33G-1)
Hong Kong, adult patient from nephrology center (carriage), 2013-2014
***Staphylococcus schleiferi* 18D12-1** (fragment of the 29 kb plasmid pT8Gsch)
South Korea, canine pyoderma
***Staphylococcus aureus* CC1-1**
France, human pus, 2009
***Enterococcus faecium* C132**
South Africa, human blood, 2019
***Staphylococcus warneri* Ani-LG-025**
Belgium, milk sample from lactating dairy cow, 2016
***Staphylococcus epidermidis* AMT** (fragment of the 24 kb plasmid pAMT2)
USA, human skin and mucous membranes, 2017
***Staphylococcus argenteus* NCTC13711**
Australia, human blood, 2006
***Staphylococcus massiliensis* P3**
Germany, human blood, 2020
***Enterococcus faecalis* C32**
South Africa, human blood, 2019
***Staphylococcus capitis* 15-101**
China, human blood, 2015
***Enterococcus faecalis* C27**
South Africa, human blood, 2019
***Staphylococcus hominis* LRKNS031**
Germany, human colonization (easy flow), 2013
***Listeria monocytogenes* LM201**
China, food, 2012
***Staphylococcus cohnii* DE0552**
USA, environmental, 2018
***Streptococcus pneumoniae* GPSC578**
Malawi, human blood, 2005
***Shinella curvata* JCM 31239**
China, desert, 2019
***Providencia alcalifaciens* wls1912**
Poland
***Acinetobacter baumannii* 22404448**
USA, human, 2021
***Mycobacterium abscessus* PAP162**
UK, human sputum, 2010
***Shigella flexneri* 126020**
UK, human, 2012

B

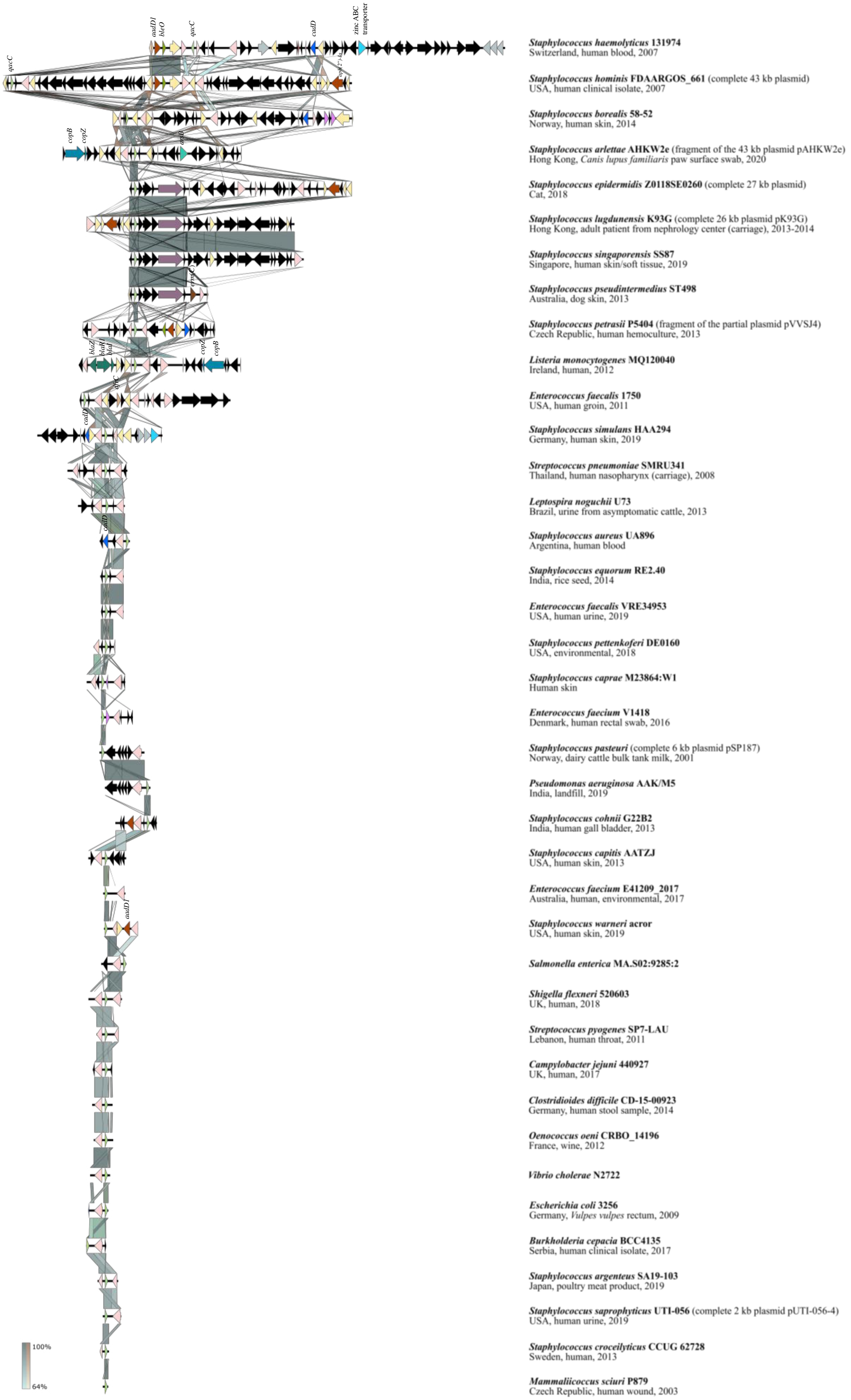
aadD1
bleO
qacC
cadD
zinc ABC transporter
qacC
copB
copZ
arsB
ermC
blaZ
blaR1
blaI
copZ
copB
dfrC
cadD
cadD
aadD1
100%
64%
Staphylococcus haemolyticus 131974
Switzerland, human blood, 2007
Staphylococcus hominis FDAARGOS_661 (complete 43 kb plasmid)
USA, human clinical isolate, 2007
Staphylococcus borealis 58-52
Norway, human skin, 2014
Staphylococcus arlettae AHKW2e (fragment of the 43 kb plasmid pAHKW2e)
Hong Kong, *Canis lupus familiaris* paw surface swab, 2020
Staphylococcus epidermidis Z0118SE0260 (complete 27 kb plasmid)
Cat, 2018
Staphylococcus lugdunensis K93G (complete 26 kb plasmid pK93G)
Hong Kong, adult patient from nephrology center (carriage), 2013-2014
Staphylococcus singaporensis SS87
Singapore, human skin/soft tissue, 2019
Staphylococcus pseudintermedius ST498
Australia, dog skin, 2013
Staphylococcus petrasii P5404 (fragment of the partial plasmid pVVSJ4)
Czech Republic, human hemoculture, 2013
Listeria monocytogenes MQ120040
Ireland, human, 2012
Enterococcus faecalis 1750
USA, human groin, 2011
Staphylococcus simulans HAA294
Germany, human skin, 2019
Streptococcus pneumoniae SMRU341
Thailand, human nasopharynx (carriage), 2008
Leptospira noguchii U73
Brazil, urine from asymptomatic cattle, 2013
Staphylococcus aureus UA896
Argentina, human blood
Staphylococcus equorum RE2.40
India, rice seed, 2014
Enterococcus faecalis VRE34953
USA, human urine, 2019
Staphylococcus pettenkoferi DE0160
USA, environmental, 2018
Staphylococcus caprae M23864:W1
Human skin
Enterococcus faecium V1418
Denmark, human rectal swab, 2016
Staphylococcus pasteuri (complete 6 kb plasmid pSP187)
Norway, dairy cattle bulk tank milk, 2001
Pseudomonas aeruginosa AAK/M5
India, landfill, 2019
Staphylococcus cohnii G22B2
India, human gall bladder, 2013
Staphylococcus capitis AATZJ
USA, human skin, 2013
Enterococcus faecium E41209_2017
Australia, human, environmental, 2017
Staphylococcus warneri acror
USA, human skin, 2019
Salmonella enterica MA.S02:9285:2
Shigella flexneri 520603
UK, human, 2018
Streptococcus pyogenes SP7-LAU
Lebanon, human throat, 2011
Campylobacter jejuni 440927
UK, human, 2017
Clostridioides difficile CD-15-00923
Germany, human stool sample, 2014
Oenococcus oeni CRBO_14196
France, wine, 2012
Vibrio cholerae N2722
Escherichia coli 3256
Germany, *Vulpes vulpes* rectum, 2009
Burkholderia cepacia BCC4135
Serbia, human clinical isolate, 2017
Staphylococcus argenteus SA19-103
Japan, poultry meat product, 2019
Staphylococcus saprophyticus UTI-056 (complete 2 kb plasmid pUTI-056-4)
USA, human urine, 2019
Staphylococcus croceilyticus CCUG 62728
Sweden, human, 2013
Mammaliicoccus sciuri P879
Czech Republic, human wound, 2003

C

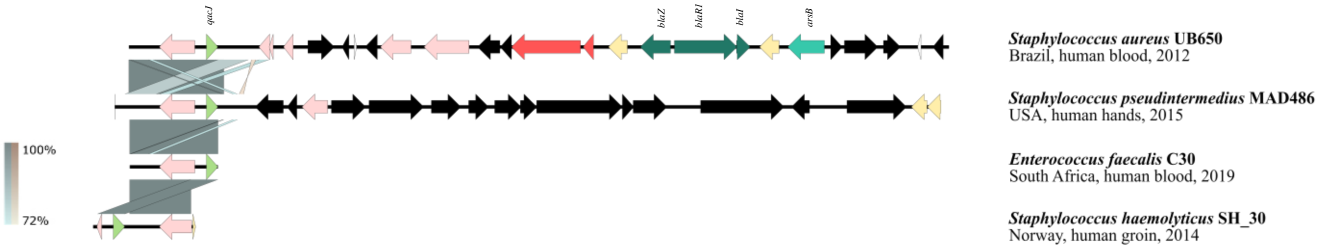
qacJ
blaZ
blaR1
blaI
arsB
100%
72%
Staphylococcus aureus UB650
Brazil, human blood, 2012
Staphylococcus pseudintermedius MAD486
USA, human hands, 2015
Enterococcus faecalis C30
South Africa, human blood, 2019
Staphylococcus haemolyticus SH_30
Norway, human groin, 2014

D

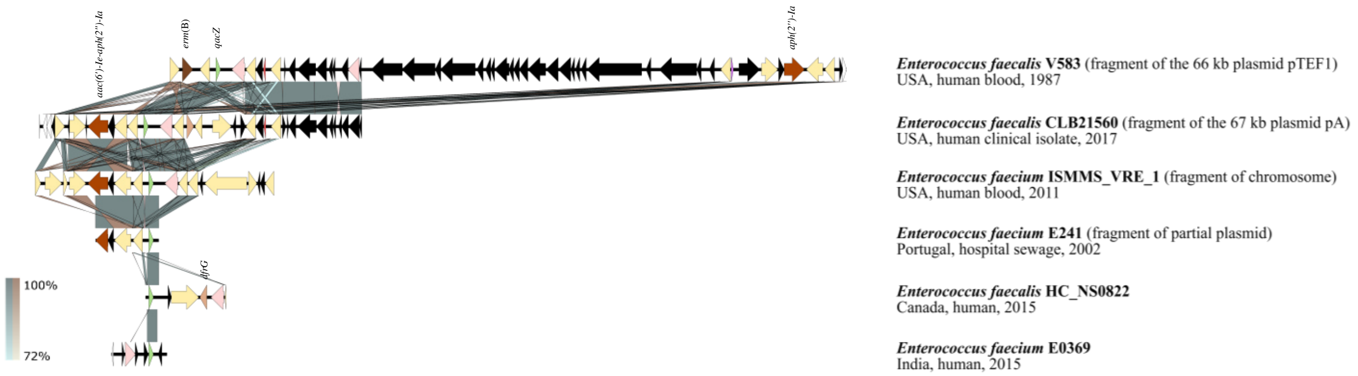
aac(6')-Ie-aph(2'')-Ia
erm(B)
qacZ
aph(2'')-Ia
dfrG
100%
72%
Enterococcus faecalis V583 (fragment of the 66 kb plasmid pTEF1)
USA, human blood, 1987
Enterococcus faecalis CLB21560 (fragment of the 67 kb plasmid pA)
USA, human clinical isolate, 2017
Enterococcus faecium ISMMS_VRE_1 (fragment of chromosome)
USA, human blood, 2011
Enterococcus faecium E241 (fragment of partial plasmid)
Portugal, hospital sewage, 2002
Enterococcus faecalis HC_NS0822
Canada, human, 2015
Enterococcus faecium E0369
India, human, 2015

E

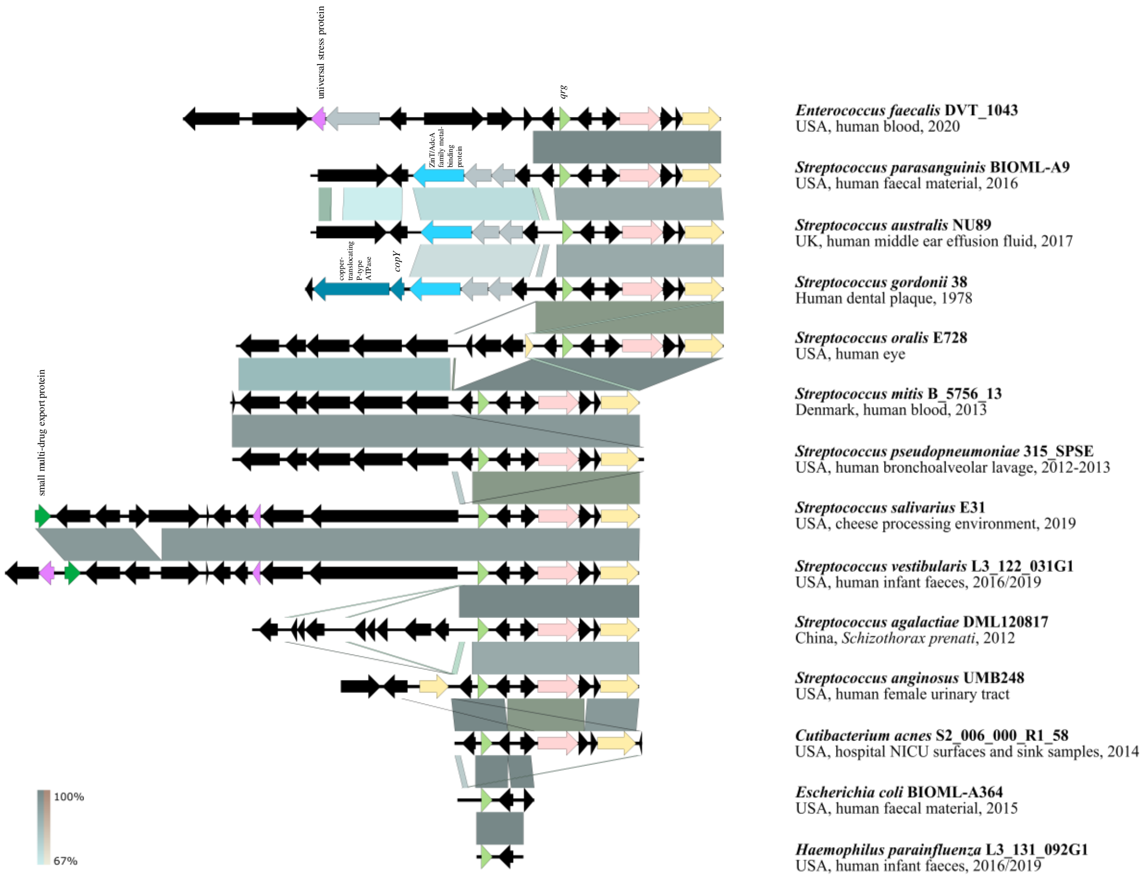
universal stress protein
qrg
ZinT/AdcA family metal-binding protein
copper-translocating P-type ATPase
copY
small multi-drug export protein
100%
67%
Enterococcus faecalis DVT_1043
USA, human blood, 2020
Streptococcus parasanguinis BIOML-A9
USA, human faecal material, 2016
Streptococcus australis NU89
UK, human middle ear effusion fluid, 2017
Streptococcus gordonii 38
Human dental plaque, 1978
Streptococcus oralis E728
USA, human eye
Streptococcus mitis B_5756_13
Denmark, human blood, 2013
Streptococcus pseudopneumoniae 315_SPSE
USA, human bronchoalveolar lavage, 2012-2013
Streptococcus salivarius E31
USA, cheese processing environment, 2019
Streptococcus vestibularis L3_122_031G1
USA, human infant faeces, 2016/2019
Streptococcus agalactiae DML120817
China, *Schizothorax prenati*, 2012
Streptococcus anginosus UMB248
USA, human female urinary tract
Cutibacterium acnes S2_006_000_R1_58
USA, hospital NICU surfaces and sink samples, 2014
Escherichia coli BIOML-A364
USA, human faecal material, 2015
Haemophilus parainfluenza L3_131_092G1
USA, human infant faeces, 2016/2019

F

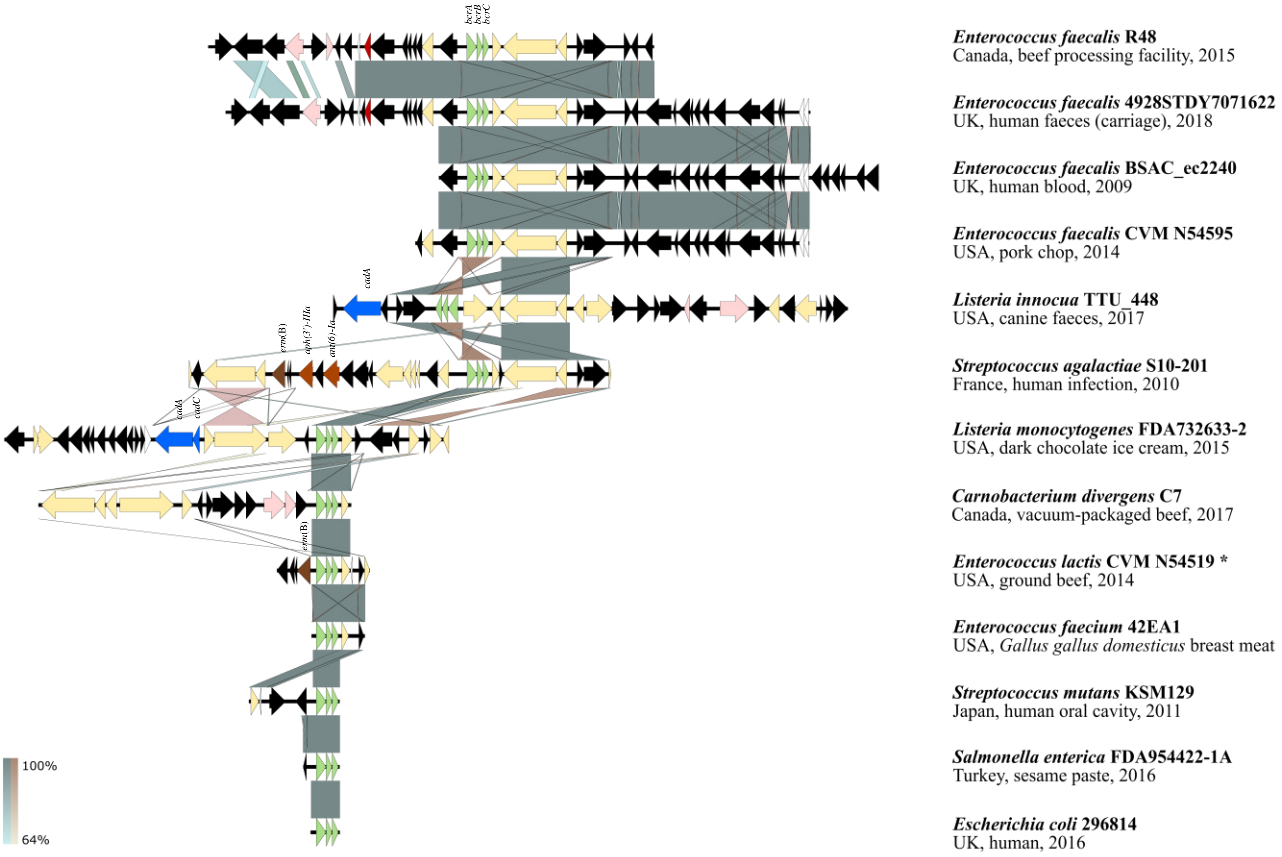
bcrA
bcrB
bcrC
cadA
erm(B)
aph(3')-IIIa
ant(6)-Ia
cadA
cadC
erm(B)
100%
64%
Enterococcus faecalis **R48**
Canada, beef processing facility, 2015
Enterococcus faecalis **4928STDY7071622**
UK, human faeces (carriage), 2018
Enterococcus faecalis **BSAC_ec2240**
UK, human blood, 2009
Enterococcus faecalis **CVM N54595**
USA, pork chop, 2014
Listeria innocua **TTU_448**
USA, canine faeces, 2017
Streptococcus agalactiae **S10-201**
France, human infection, 2010
Listeria monocytogenes **FDA732633-2**
USA, dark chocolate ice cream, 2015
Carnobacterium divergens **C7**
Canada, vacuum-packaged beef, 2017
Enterococcus lactis **CVM N54519 ***
USA, ground beef, 2014
Enterococcus faecium **42EA1**
USA, *Gallus gallus domesticus* breast meat
Streptococcus mutans **KSM129**
Japan, human oral cavity, 2011
Salmonella enterica **FDA954422-1A**
Turkey, sesame paste, 2016
Escherichia coli **296814**
UK, human, 2016

G

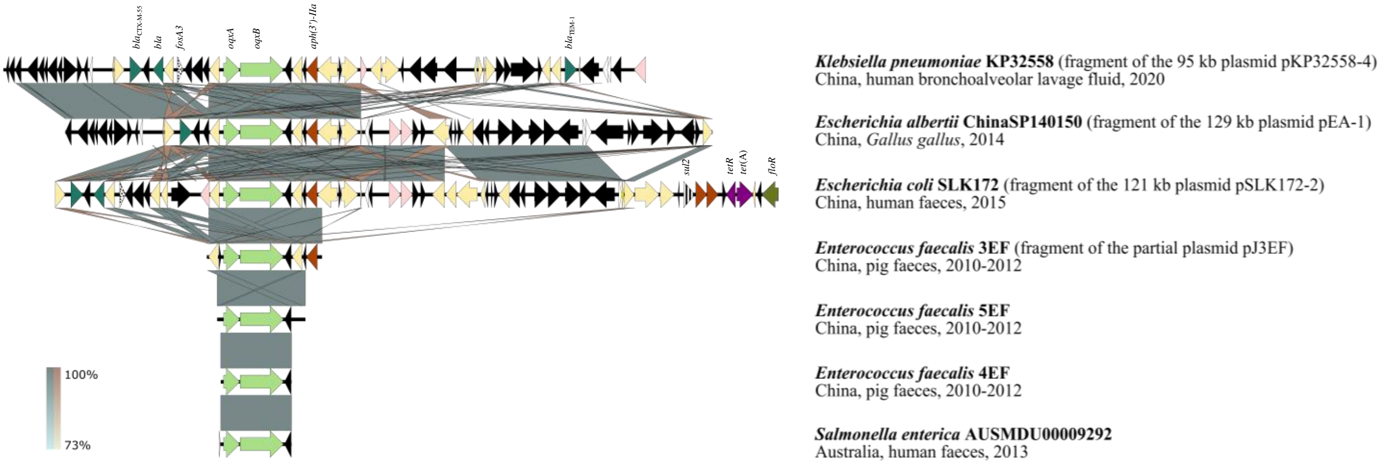
$bla_{CTX-M-55}$
bla
fosA3
oqxA
oqxB
aph(3')-IIIa
bla_{TEM-1}
sul2
tetR
tet(A)
floR
100%
73%
Klebsiella pneumoniae **KP32558** (fragment of the 95 kb plasmid pKP32558-4)
China, human bronchoalveolar lavage fluid, 2020
Escherichia albertii **ChinaSP140150** (fragment of the 129 kb plasmid pEA-1)
China, *Gallus gallus*, 2014
Escherichia coli **SLK172** (fragment of the 121 kb plasmid pSLK172-2)
China, human faeces, 2015
Enterococcus faecalis **3EF** (fragment of the partial plasmid pJ3EF)
China, pig faeces, 2010-2012
Enterococcus faecalis **5EF**
China, pig faeces, 2010-2012
Enterococcus faecalis **4EF**
China, pig faeces, 2010-2012
Salmonella enterica **AUSMDU00009292**
Australia, human faeces, 2013

Antimicrobials

Quaternary ammonium compounds tolerance associated genes

Antibiotic resistance: Aminoglicosides Beta-lactams Bleomycin Chloramphenicol/Florfenicol Fosfomycin

Macrolides, Lincosamides and/or Streptogramin B Mupirocin Sulfonamides Tetracyclines

Trimethoprim

Metal tolerance: Arsenic Cadmium Copper Zinc Metal transporter

Bacteriocin associated

Multiple functions

UV resistance Toxin-Antitoxin system Stress response Insertion sequence/Recombinase/Integrase/Transposase

Replication associated

Fig S1 Diversity of genetic contexts among *Enterococcus* and other bacterial taxa genomes of QACs tolerance genes: A*) qacA/B*; B) *qacC*; C) *qacJ*; D) *qacZ*; E) *qrg*; F) *bcrABC*; G) *oqxAB.* For each gene, *Enterococcus* strains representative of the different sources available were selected as well as one genome corresponding to each of the remaining species, from several sources and timespans. Images were generated using the BLASTN option of Easyfig 2.2.2 (82) and based on NCBI genome annotations.

* For *Enterococcus* strain CVM N54519 the best-matching type-strain, *Enterococcus lactis,* was considered.

Table S2 - Epidemiological background of the 105 *E. faecium* and 105 *E. faecalis* included in the QACs susceptibility phenotypic assays.

Species	Origin (n isolates)	Source (n isolates)	Date	Country (n isolates)	ST (n isolates)	CT (n isolates)	MDR
Enterococcus faecium n=105	Humans n=45	Human infection: diverse biological products (26)	1996-2020	Portugal (20), Spain (3), Tunisia (3)	17 (2), 18 (3), 22 (2), 78 (1), 80 (2), 117 (3), 190 (1), 280 (2), 391 (1), 412 (2), 419 (1), 494 (1), 515 (1), 780 (1), NA (3)	24 (1), 222 (1), 258 (1), 730 (1), 948 (1), 962 (2), 1764 (1), 4639 (1), 4643 (1), 4651 (1), 4652 (1), 4658 (1), 4659 (1), 4660 (1), 4719 (1), 5005 (1), NA (9)	92%
		Healthy humans colonization: faeces (14), vagina (1)	2001-2017	Portugal (10), Angola (2), Unknown (3)	6 (2), 8 (1), 18 (1), 22 (1), 32 (1), 89 (1), 147 (1), 845 (2), 1051 (1), 2095 (1), NA (3)	124 (1), 128 (1), 4641 (1), 4647 (1), 4672 (1), NA (10)	73%
		Long-term care patients: faeces (4)	2015-2016	Portugal (4)	80 (2), 117 (1), 262 (1)	24 (1), NA (3)	100%
	Food chain. n=49	Animal production settings: aquaculture (5), piggery (10), poultry faeces (3)	2001-2020	Portugal (11), Angola (3), Unknown (4)	5 (1), 6 (2), 29(1), 30 (1), 94 (1), 101 (1), 132 (1), 185 (1), 264 (1), 393 (1), 430 (1), 434 (1), 891 (1), 970 (1), 971 (1), NA (2)	128 (2), 374 (1), 549 (1), 749 (1), 967 (1), 4644 (1), 4645 (1), 4664 (1), 4665 (1), 4666 (1), 4667 (1), NA (6)	61%
		Food of animal origin: poultry carcass (10), trout (5), bovine milk (4), bovine meat (3), raw meat frozen pet food (5)	1999-2020	Portugal (18), Tunisia (9)	9 (2), 12 (1), 17 (2), 18 (2), 25 (1), 27 (1), 32 (1), 80 (1), 157 (1), 203 (1), 245 (1), 264 (1), 451 (1), 683 (1), 1058 (2), 1059 (1), 1091 (1), 1263 (1), 2092 (1), NA (4)	106 (1), 184 (1), 222 (1), 272 (1), 284 (1), 374 (1), 948 (2), 2661 (1), 3399 (1), 4638 (2), 4653 (1), 4654 (1), 4655 (1), 4656 (1), 4657 (1), 4668 (1), 4669 (1), 4670 (1), NA (7)	39%
		Ready-to-eat salads (4)	2010	Portugal (4)	352 (1), 640 (2), 666 (1)	4662 (1), 4663 (1), NA (2)	0%
	Wild birds n=2	Wild birds (2)	2011-2013	Germany (1), Canada (1)	78 (1), 448 (1)	63 (1), 4642 (1)	100%
	Aquatic environment n=9	River (3)	2003	Portugal (3)	22 (1), 132 (1), 2093 (1)	5140 (1), NA (2)	67%
		Hospital sewage (3)	2001-2002	Portugal (3)	17 (1), 368 (1), 2091 (1)	4649 (1), NA (2)	67%
		Urban wastewater treatment plant (3)	2014-2015	Tunisia (3)	666 (1), NA (2)	NA (3)	67%
Enterococcus faecalis n=105	Humans n=55	Human infection: diverse biological products (27)	1987-2020	Portugal (22), Tunisia (4), USA (1)	2 (1), 6 (6), 9 (1), 16 (3), 22 (1), 25 (1), 30 (1), 55 (1), 59 (1), 64 (1), 116 (1), 133 (1), 159 (1), 191 (1), 200 (1), 631 (1), 1105 (1), 1165 (1), NA (2)	1248 (3), 1252 (1), 1265 (1), 1266 (1), 1267 (1), 1271 (1), 1376 (1), 1408 (1), 1431 (1), 1438 (1), 1442 (1), 1463 (1), 1471 (1), 1475 (1), 1481 (1), 1486 (1), 2904 (1), 2905 (1), 2906 (1), 2907 (1), NA (5)	59%
		Healthy humans colonization: faeces (14), urinary tract (6), breast milk (1)	2001-2018	Portugal (19), Angola (2)	21 (1), 30 (1), 40 (6), 63 (1), 64 (1), 81 (1), 116 (1), 179 (2), 191 (1), 200 (1), 206 (1), 209 (1), 275 (1), 308 (1), 394 (1)	1219 (1), 1226 (1), 1232 (1), 1293 (1), 1294 (1), 1295 (1), 1309 (1), 1311 (1), 1319 (1), 1320 (1), 1323 (1), 1325 (1), 1328 (1), 1340 (1), 1343 (1), 1348 (1), 1380 (1), 1489 (1), 1493 (1), 1567 (1), 1665 (1)	48%
		Long-term care patients: faeces (4)	2015-2016	Portugal (4)	25 (1), 143 (1), 398 (1), 679 (1)	1364 (1), 1366 (1), 1368 (1), 1372 (1)	25%
		Patients at hospital admission: faeces (3)	2015-2016	Brasil (3)	6 (2), 525 (1)	1296 (2), 1298 (1)	100%
	Food chain n=40	Animal production settings: aquaculture (5), piggery (7), poultry and ovine faeces (2)	2006-2015	Portugal (12), Tunisia (3)	21 (1), 22 (1), 35 (1), 40 (1), 59 (1), 65 (1), 139 (1), 200 (1), 209 (1), 330 (1), 386 (1), 631 (1), 749 (1), 872 (1)	1246 (1), 1262 (1), 1439 (1), 1505 (1), 1506 (1), 1513 (1), 1520 (1), 1524 (1), 1537 (1), 1548 (1), 1549 (1), 1556 (1), 1528 (1), 1534 (1)	36%
		Food of animal origin: poultry carcass (7), trout (6), bovine and goat milk (3), bovine meat (2), raw meat frozen pet food (4)	1999-2020	Portugal (17), Tunisia (5)	21 (1), 27 (1), 32 (1), 34 (1), 40 (1), 49 (1), 117 (1), 141 (1), 200 (1), 202 (1), 206 (1), 209 (1), 227 (1), 288 (1), 436 (1), 674 (1), 721 (1), 843 (1), 860 (1), 1008 (2), 1106 (1)	1205 (2), 1206 (1), 1207 (1), 1240 (1), 1243 (1), 1269 (1), 1275 (1), 1288 (1), 1292 (1), 1557 (1), 1558 (1), 1559 (1), 1563 (1), 1564 (1), 1565 (1), 1571 (1), 1583 (1), 1589 (1), 1600 (1), 1629 (1), 1640 (1)	36%
		Ready-to-eat salads (4)	2010	Portugal (4)	141 (1), 165 (1), 309 (1), 594 (1)	1496 (1), 1497 (1), 1498 (1), 1499 (1)	0%
	Pet faeces n=2	Pet faeces: cat (1), bird (1)	2014-2015	Tunisia (2)	21 (1), 116 (1)	1260 (1), 1264 (1)	0%
	Aquatic environment. n=8	River (2)	2003	Portugal (2)	1 (1), 4 (1)	1 (1), 1467 (1)	0%
		Hospital sewage (3)	2002	Portugal (3)	16 (1), 35 (1), 49 (1)	1656 (1), 1658 (1), 1659 (1)	100%
		Urban wastewater treatment plant (3)	2014-2015	Tunisia (3)	23 (1), 86 (1), 117 (1)	20 (1), 1241 (1), 1244 (1)	33%

Abbreviations: CT, complex type; MDR, multidrug resistance; n, number; NA, not available; QACs, quaternary ammonium compounds; ST, sequence type; USA, United States of America.

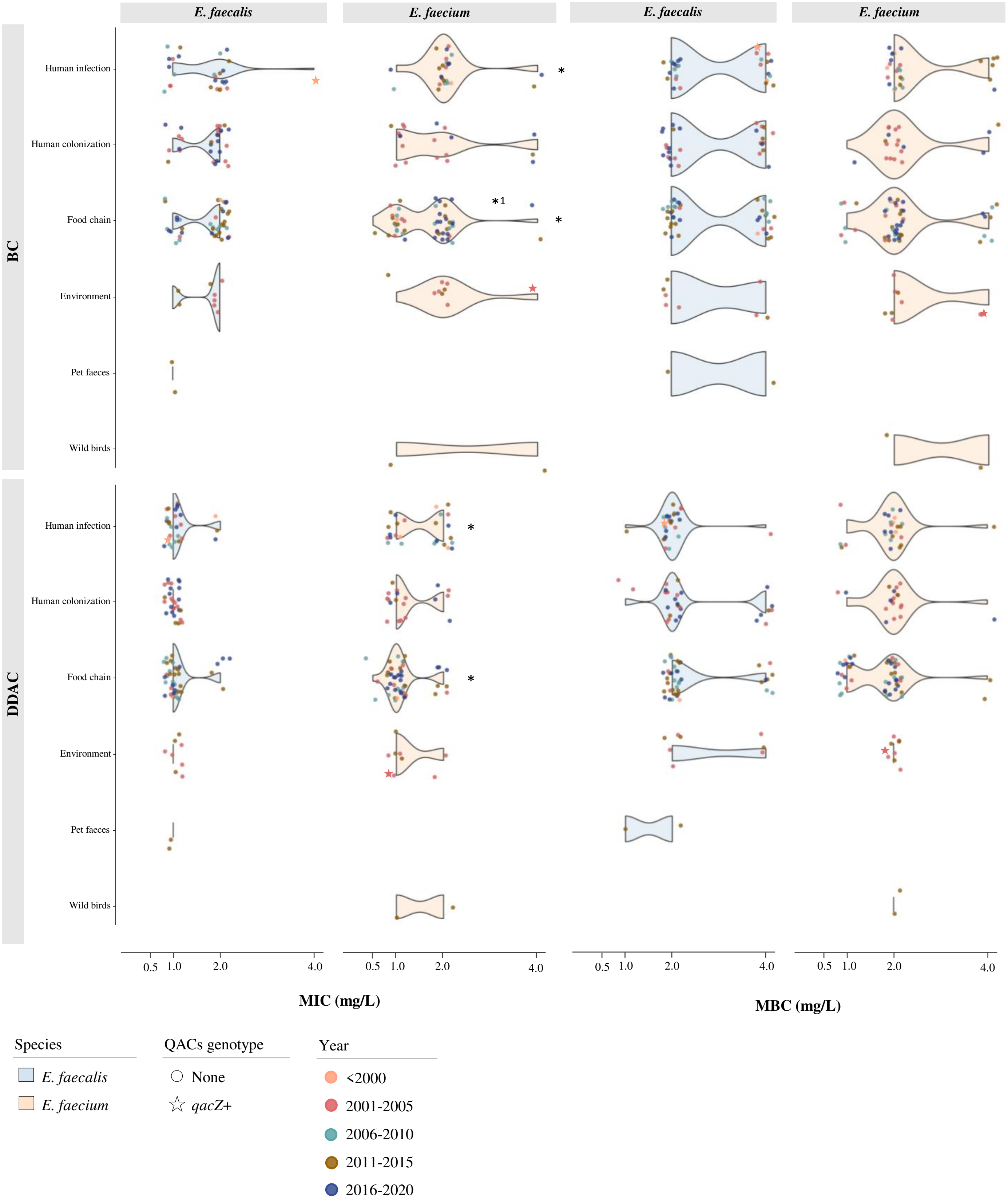

Fig S2 Benzalkonium chloride (BC) and didecyldimethylammonium chloride (DDAC) minimum inhibitory concentrations (MIC) and minimum bactericidal concentrations (MBC) distribution of *Enterococcus faecalis* (blue) and *Enterococcus faecium* (orange) isolates from independently analysed sources. The isolation date and QACs genotype of each isolate is indicated with different colours and shapes, respectively. For more isolates' details see Table S2. * MIC_{DDAC} and MIC_{BC} were higher for *E. faecium* recovered from human infections compared to those from the food chain ($P \leq 0.01$). *[1] MIC_{BC} significantly increased over the years in isolates from the food chain ($P \leq 0.01$). Graphics were done in R, using the ggplot2 package v3.4.0 (35).

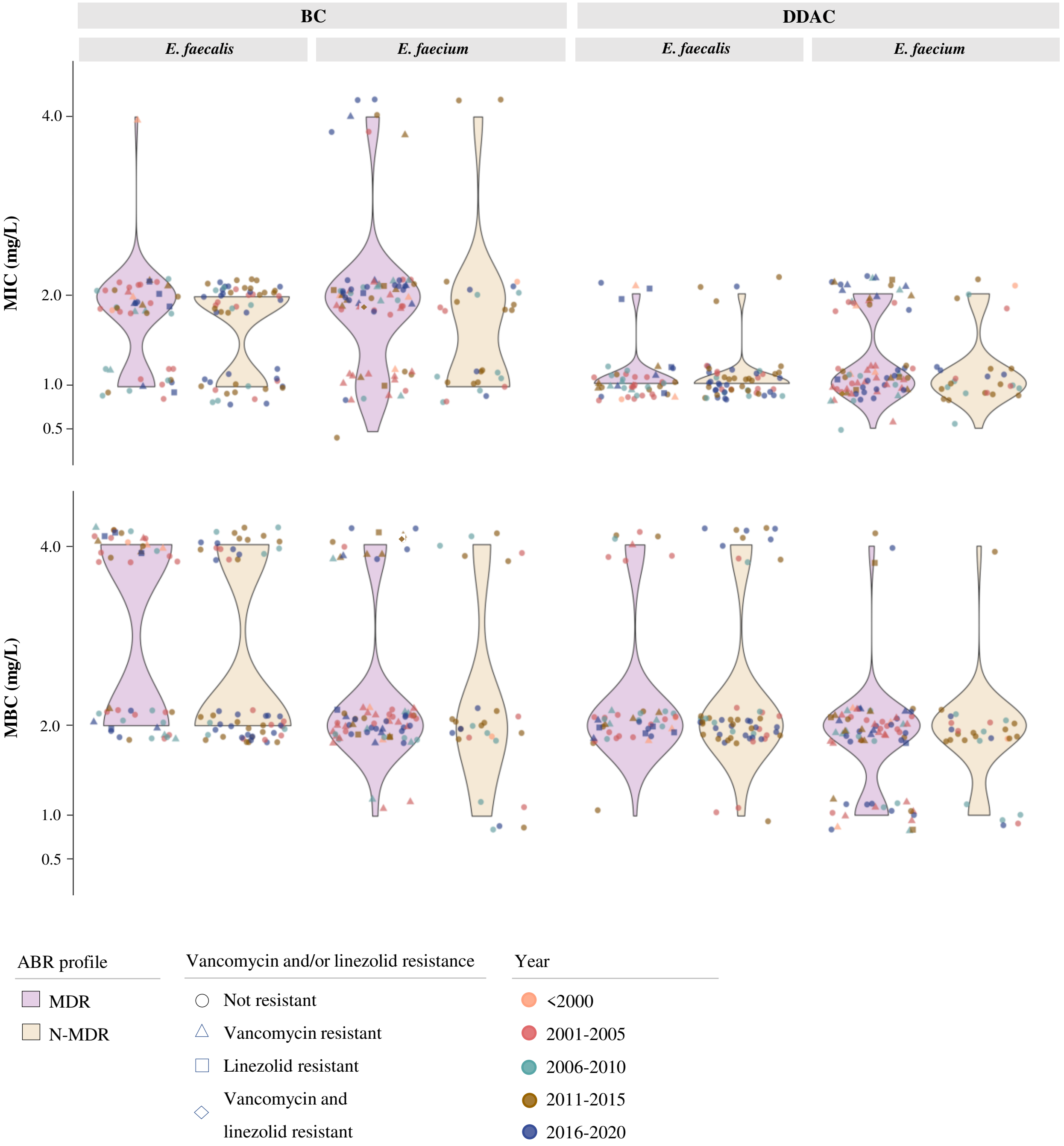

Fig S3 Benzalkonium chloride (BC) and Didecyldimethylammonium chloride (DDAC) minimum inhibitory concentrations (MIC) and minimum bactericidal concentrations (MBC) distribution of *Enterococcus faecalis* and *Enterococcus faecium* multidrug resistant (MDR; pink) and non-multidrug resistant (N-MDR; beige) isolates. The isolation date of each isolate and its resistance to vancomycin and/or linezolid is indicated with different colours and shapes, respectively. For more isolates' details see Table S2. Graphics were done in R, using the ggplot2 package v3.4.0 (35).

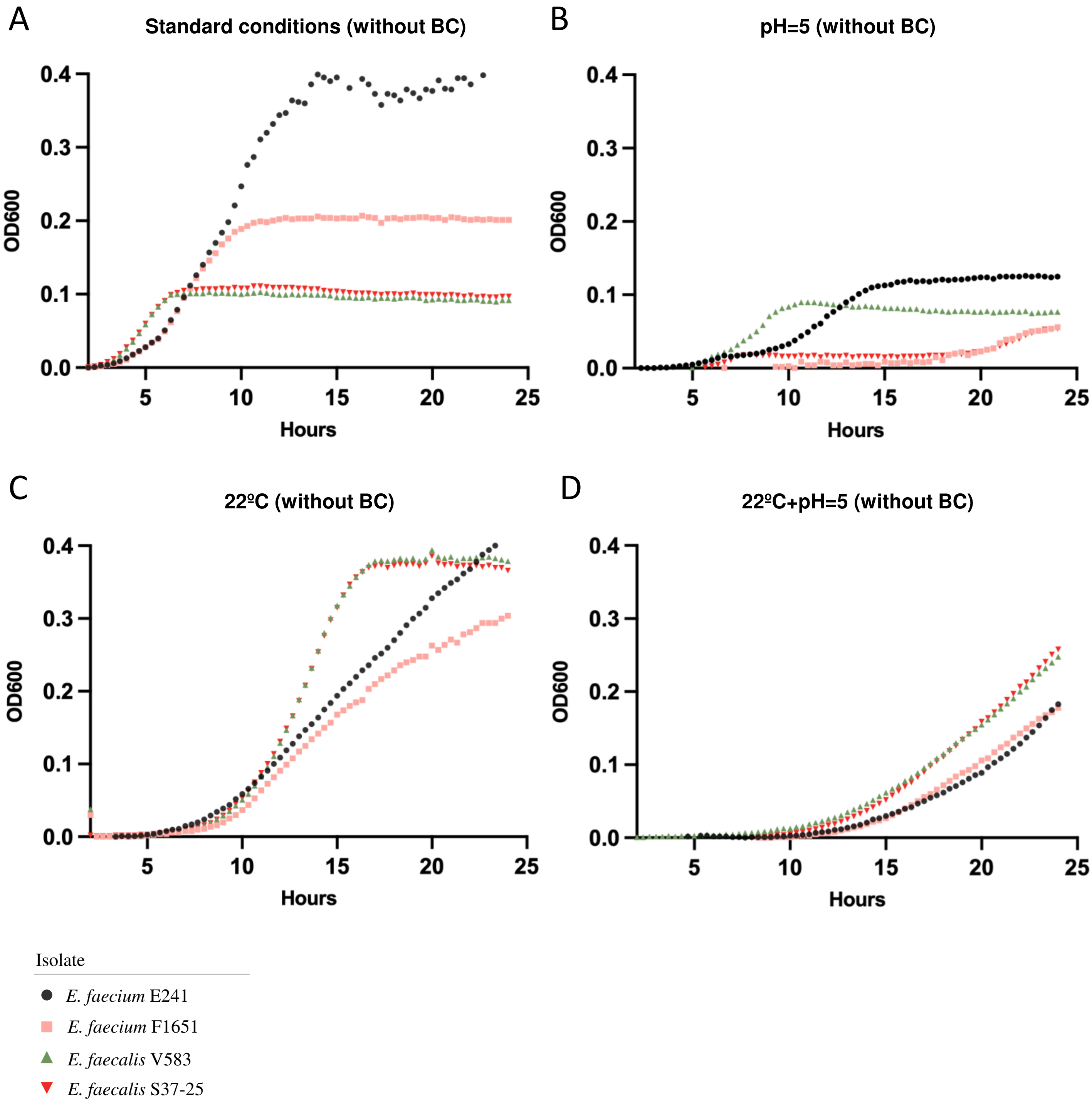

Fig S4 Growth kinetics without benzalkonium chloride (BC) for *E. faecium* E241 (black circles), *E. faecium* F1651 (pink squares), *E. faecalis* V583 (green triangles) and *E. faecalis* S37-25 (inverted red triangles) in standard (A) or modified conditions (B: pH=5; C: 22ºC; D: 22ºC+pH=5). For more isolates' details see Table S3. Growth curves were determined using a Biotek Synergy HT plate reader (Marshall Scientific), with absorbance at 600 nm (A600) recorded every 20 minutes for 24 hours. Graphics were built using the Prism software v8.1.1 (GraphPad Software; www.graphpad.com).

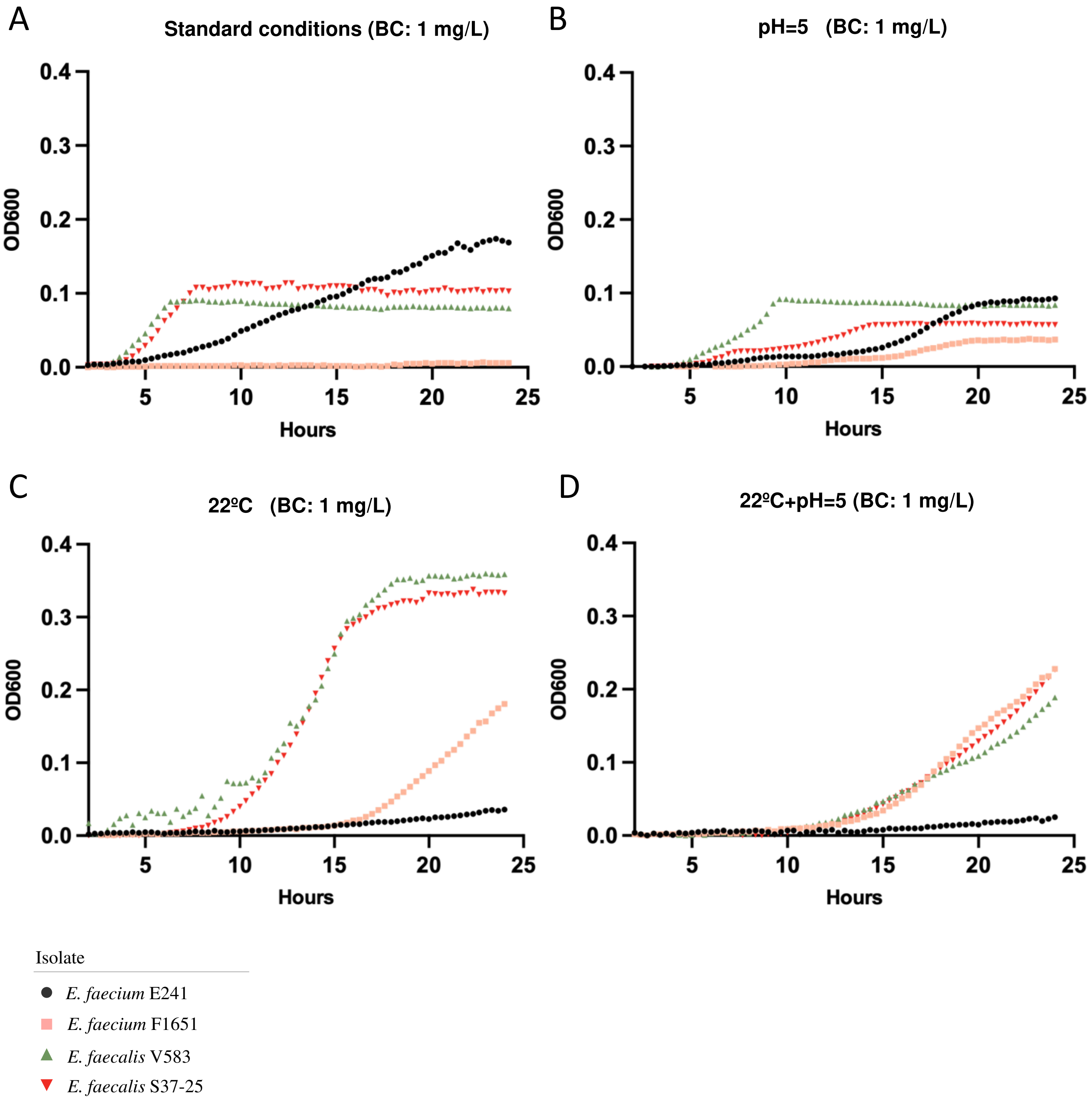

Fig S5 Growth kinetics in the presence of 1 mg/L of benzalkonium chloride (BC) for *E. faecium* E241 (black circles), *E. faecium* F1651 (pink squares), *E. faecalis* V583 (green triangles) and *E. faecalis* S37-25 (inverted red triangles) in standard (A) or modified conditions (B: pH=5; C: 22ºC; D: 22ºC+pH=5). For more isolates' details see Table S3. Growth curves were determined using a Biotek Synergy HT plate reader (Marshall Scientific), with absorbance at 600 nm (A600) recorded every 20 minutes for 24 hours. Graphics were built using the Prism software v8.1.1 (GraphPad Software; www.graphpad.com).

TABLE S3 – Epidemiological background of the 22 *E. faecalis* and 20 *E. faecium* included in the modified QACs susceptibility phenotypic assays.

Species	Isolate	ST	CT	Date	Isolation Source	Country	Antibiotic Resistance Profile	Modified conditions tested			
								Anaerobiosis	22ºC	pH5	22ºC + pH5
E. faecium	M239495	412	258	2011	Human infection	Portugal	AMP, CIP, ERY, STR, TET	●	●	●	
	342T	18	222	2014	Human infection	Tunisia	AMP, CIP, ERY, STR	●	●	●	
	HPH109	494	NA	2020	Human infection	Portugal	AMP, CIP, ERY, GEN, STR, TET, TGC	●	●	●	●
	TR 58_34	683	4669	2012	Trout	Portugal	TET	●	●	●	
	R7	22	NA	2003	River	Portugal	CIP	●	●	●	●
	E241 *	17	NA	2002	Hospital sewage	Portugal	AMP, CIP, ERY, GEN, Q-D, STR, TET	●	●	●	●
	66T	666	NA	2015	Urban wastewater treatment plant	Tunisia	CIP, ERY, STR, TET	●	●	●	
	S4_1E	640	4663	2010	Ready-to-eat salad	Portugal	-	●	●	●	
	S53_34E	352	NA	2010	Ready-to-eat salad	Portugal	-	●	●	●	●
	D1	147	NA	2003	Human colonization (faeces)	Unknown	ERY, STR, TEC, TET, VAN	●	●	●	
	A29.1A	1051	NA	2015	Human colonization (faeces)	Angola	Q-D	●	●	●	
	C75	89	NA	2001	Human colonization (faeces)	Portugal	ERY, Q-D, STR, TEC, VAN	●	●	●	●
	c29VSb_20_AE	8	NA	2017	Human colonization (vagina)	Portugal	ERY, TET	●	●	●	
	CCT11	117	24	2015-2016	Long-term care patient (faeces)	Portugal	AMP, CIP, ERY, TEC, VAN	●	●	●	
	CCT96	262	NA	2015-2016	Long-term care patient (faeces)	Portugal	AMP, CIP, ERY	●	●	●	●
	D2	6	128	2001	Piggery	Unknown	AMP, ERY, TEC, TET, VAN	●	●	●	
	F1651	12	NA	2019	Poultry carcass	Portugal	ERY, Q-D, STR, TET	●	●	●	●
	145T	1058	4638	2015	Bovine milk	Tunisia	CIP, ERY, LZD, Q-D, TET	●	●	●	
	4275	448	4642	2011	Wild bird	Germany	AMP, CIP, ERY, STR, TEC, TET, VAN	●	●	●	
	PF97	264	374	2019	Raw meat frozen pet food	Portugal	AMP, CIP, Q-D, STR, TET	●	●	●	
E. faecalis	V583 *	6	371	1987	Human infection	USA	ERY, GEN, VAN	●	●	●	●
	204070	159	1252	1999	Human infection	Portugal	CIP, ERY, GEN, TEC, TET, VAN	●	●	●	●
	H307	9	1442	2002	Human infection	Portugal	CHL, CIP, ERY, GEN, STR, TEC, VAN	●	●	●	
	HPH25	191	2905	2019	Human infection	Portugal	TET	●	●	●	
	M5366845	6	2907	2013	Human infection	Portugal	CIP, ERY, GEN, STR, TET	●	●	●	
	TR 39-20	139	1549	2011	Aquaculture	Portugal	ERY, TET	●	●	●	
	R8	4	1467	2003	River	Portugal	-	●	●	●	●
	E263	16	1656	2002	Hospital sewage	Portugal	ERY, GEN, TET, STR	●	●	●	
	14T	23	1241	2014	Urban wastewater treatment plant	Tunisia	TET	●	●	●	
	S37-25E	309	1499	2010	Ready-to-eat salad	Portugal	-	●	●	●	●
	C72	40	1226	2001	Human colonization (faeces)	Portugal	CHL, ERY, STR, TET	●	●	●	
	C370	63	1340	2001	Human colonization (faeces)	Portugal	CHL, ERY, STR, TET	●	●	●	
	A35_1A	81	1294	2013	Human colonization (faeces)	Angola	ERY, TET	●	●	●	
	Br16	525	1298	2015	Human colonization (faeces)	Brazil	GEN, TET	●	●	●	●
	c20Ua_66_AE	200	1320	2017	Human colonization (urine)	Portugal	CIP, ERY, GEN, TEC, TET, VAN	●	●	●	
	CCM48	25	1366	2015-2016	Long-term care patient (faeces)	Portugal	STR	●	●	●	
	SN163	22	1505	2006-2007	Piggery	Portugal	CHL, ERY, MIN, STR, TET	●	●	●	
	F1155	843	1571	2018	Poultry carcass	Portugal	ERY, TET	●	●	●	●
	138T	117	1243	2015	Bovine milk	Tunisia	-	●	●	●	
	273T	872	1262	2015	Ovine faeces	Tunisia	-	●	●	●	
	PF63	40	1206	2019	Raw meat frozen pet food	Portugal	CHL, ERY, LZD, TET	●	●	●	
	PF110	674	1207	2019	Raw meat frozen pet food	Portugal	CHL, CIP, ERY, LZD, STR, TET	●	●	●	●

* *qacZ+*

\- not resistant to the antibiotics tested

Abbreviations: AMP, ampicillin; CHL, chloramphenicol; CIP, ciprofloxacin; CT, complex type; ERY, erythromycin; GEN, gentamicin; LZD, linezolid; MIN, minocycline; QACs, quaternary ammonium compounds; Q-D, quinupristin-dalfopristin; ST, sequence type; STR, streptomycin; TEC, teicoplanin; TET, tetracycline; TGC, tigecycline; USA, United States of America.

Table S4 - Benzalkonium chloride neutralizer* effectiveness and toxicity test.

**Strain**	**Test solution**	**Bacterial cell count (CFU/mL)** [1]
Enterococcus faecalis ATCC29212	NaCl 0.9 % (Control)	3.2×10^5
		1.8×10^5
		2.1×10^5
	Neutralizer* + 2 mg/L of Benzalkonium chloride [2]	2.8×10^5
		3.3×10^5
		2.4×10^5
	NaCl 0.9 % + 2 mg/L of Benzalkonium chloride [2]	0.4×10^5
		0.1×10^5
		0.1×10^5
Enterococcus faecalis V583	NaCl 0.9 % (Control)	2.3×10^5
		3.2×10^5
		1.7×10^5
	Neutralizer* + 4 mg/L of Benzalkonium chloride [2]	2.7×10^5
		4.5×10^5
		3.0×10^5
	NaCl 0.9 % + 4 mg/L of Benzalkonium chloride [2]	Absence of colonies
		Absence of colonies
		Absence of colonies

* The neutralizer solution was prepared according to EN 13727 (ref) and contained Tween 80 (30 g/L; CAS: 9005-65-6, Sigma Aldrich), sodium dodecyl sulfate (4 g/L; CAS: 151-21-3, VWR) and lecithin (3 g/L; MP Biomedicals).

[1] The three values presented for each test solution correspond to different replicates.

[2] Benzalkonium chloride concentrations used in these tests corresponded to the minimum bactericidal concentration (MBC) of each strain in standard conditions.
